# Supplementary material for: The Alzheimer's disease‐associated C99 fragment of APP regulates cellular cholesterol trafficking
Source: EMBO J. 2020 Aug 31;39(20):e103791. doi: 10.15252/embj.2019103791 (PMC7560219; doi:10.15252/embj.2019103791)
Supplement: Supplementary file 1 — Appendix [file EMBJ-39-e103791-s001.pdf]

APPENDIX

THE C99 FRAGMENT OF APP REGULATES CHOLESTEROL TRAFFICKING

Table of contents

1- Appendix Figure S1 ……………………………………… PAGE 2

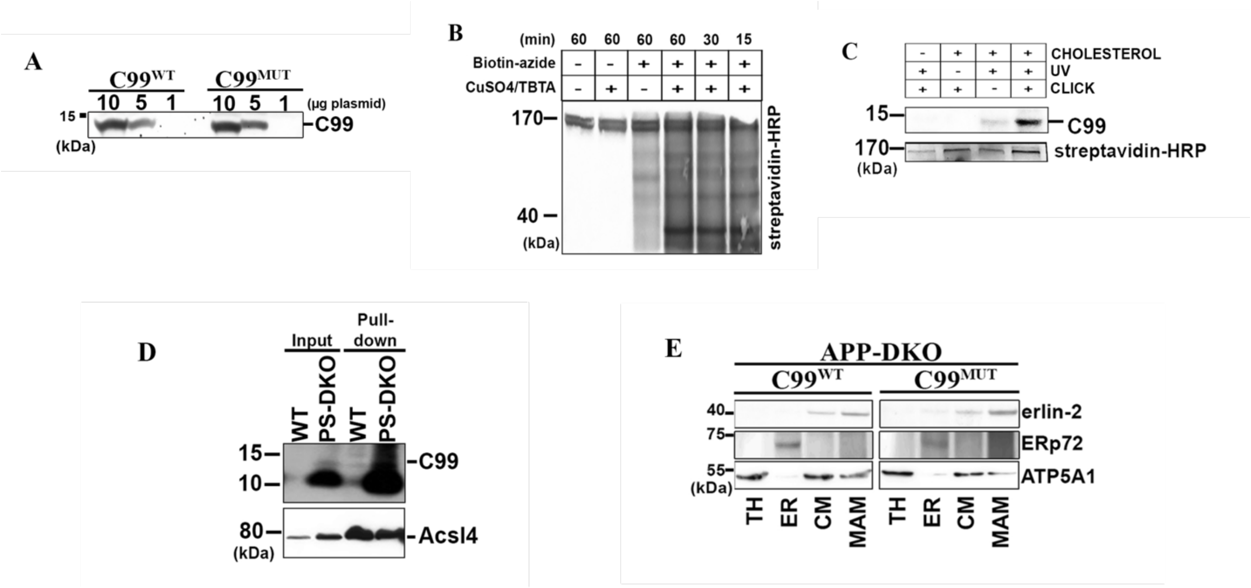

Appendix Figure S1 (A) APP-DKO cells were transfected with the indicated amounts of plasmid expressing either C99^{WT} or C99^{MUT} and their expression was detected by WB using 6E10 antibody **(B)** Biotinylation levels were detected using streptavidin-HRP to optimize CLICK chemistry conditions. Note that the specific biotinylation of PhotoClick cholesterol can be detected 15 min after initiation of the click reaction. **(C)** APP-DKO cells expressing the C99 construct were incubated with PhotoClick cholesterol and exposed to UV for crosslinking. PhotoClick cholesterol was biotinylated using CLICK chemistry before using streptavidin beads to pull down the biotinylated cholesterol. In parallel, either PhotoClick cholesterol, UV or CLICK reagents were omitted to assess the specificity of the approach. The presence of C99 interacting with the pulled-down PhotoClick cholesterol was analyzed by immunoblotting for each condition. Note that C99 levels are undetectable when either PhotoClick cholesterol, UV or CLICK reagents are not added. Streptavidin-HRP treatment was used to reveal endogenously biotinylated proteins (used as loading control). **(D)** MAM fractions from WT or PS-DKO cells incubated with PhotoClick cholesterol were used to assess if endogenous C99 was detected upon pull-down of biotinylated PhotoClick cholesterol, thus corroborating the feasibility of this technique for analyzing C99-cholesterol interaction. **(E)** Western blot of subcellular fractions isolated in Fig. 5B-D. Erlin-2, ERp72 and ATP5A1 were used as MAM, ER and crude membrane (CM) markers, respectively (TH: total homogenate).
