# Supplementary material for: The Alzheimer's disease‐associated C99 fragment of APP regulates cellular cholesterol trafficking
Source: EMBO J. 2020 Aug 31;39(20):e103791. doi: 10.15252/embj.2019103791 (PMC7560219; doi:10.15252/embj.2019103791)
Supplement: Supplementary file 2 — Expanded View Figures PDF [file EMBJ-39-e103791-s002.pdf]

Expanded View Figures

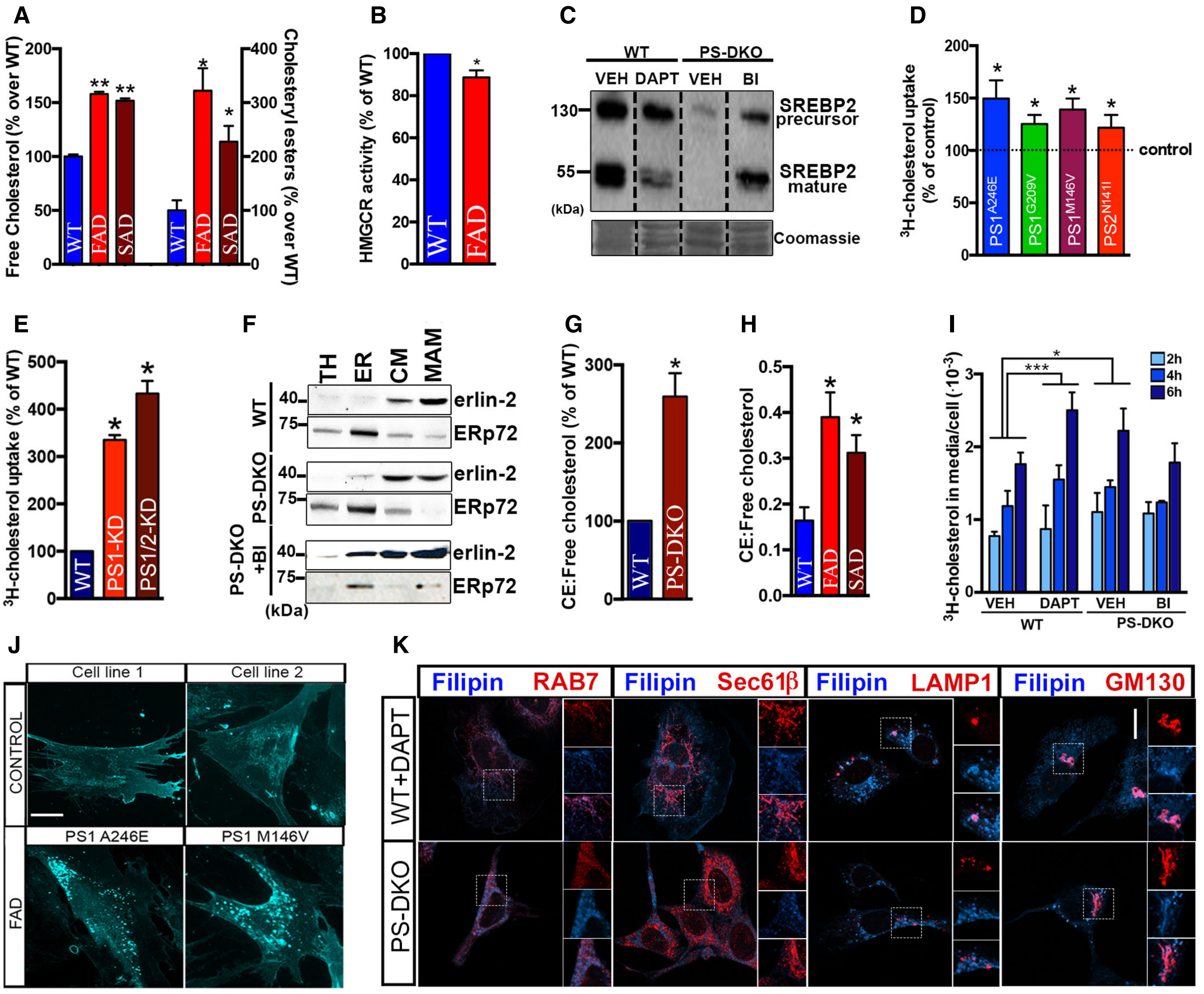

Figure EV1.

Figure EV1. Increases in MAM-localized C99 induces cholesterol internalization and trafficking.

A Quantification of free cholesterol and cholesteryl ester levels in total homogenates from fibroblasts from familial (FAD) and sporadic (SAD) AD patients by lipidomics analysis. Lipid units are represented as molar mass over total moles of lipids analyzed (mol%). Graphs represent fold change over controls (WT). Unpaired t-test vs. WT (n = 4–8; $*P < 0.05$, $**P < 0.01$).

B Quantification of HMGCR enzymatic activity showed a decreased rate of the *de novo* synthesis of cholesterol in fibroblasts from FAD patients. One-sample t-test vs. WT (n = 3–4; $*P < 0.05$).

C Measurement of SREBP2 levels by WB showed reductions in its mature/active form in DAPT-treated WT cells, and a reduction in both the full-length/precursor and mature forms in PS-DKO cells. Note how reductions in SREBP2 levels in mutant cells were abrogated upon BACE1 inhibition (BI). Coomassie staining is shown as a loading control.

D, E Quantification of cholesterol uptake in (D) FAD fibroblasts and (E) neuroblastoma cell lines (Neuro-2a) where PS1, or both PS1 and PS2, had been transiently silenced. Dashed line indicates control levels. One-sample t-test vs. WT (n = 3; $*P < 0.05$).

F Western blot of isolated MAM and ER fractions used in Fig 1E and F. Erlin-2 and ERp72 were used as MAM and ER markers, respectively (TH: total homogenate, CM: crude membrane).

G, H Ratio of cholesteryl esters (CE):free cholesterol levels measured by lipidomic analysis of (G) WT and PS-DKO cells homogenate, and in (H) AD fibroblasts. One-sample t-test vs. WT (n = 3; $*P < 0.05$).

I Cholesterol efflux, at 2, 4, and 6 h, upon 1 h pulse-chase with ^{3}H-cholesterol was assayed in WT or PS-DKO cells previously treated for 16 h with DAPT or a BACE inhibitor (BI), respectively. DMSO was used as a vehicle. Two-way ANOVA (Time, Group) (n = 3; $*P < 0.05$, $***P < 0.001$).

J Endogenous levels of cholesterol were detected by filipin staining in the indicated FAD cell lines and age-matched controls. Scale bar = 20 μm.

K Representative confocal images of PS-DKO and DAPT-treated WT cells where the distribution of free cholesterol was analyzed by co-staining with filipin (blue) and the indicated markers (labeled in red) of endosomes (Rab7), ER (Sec61β), lysosomes (LAMP1), and Golgi (GM130). Scale bar = 20 μm. Zoom images are 5×.

Source data are available online for this figure.

 © 2020 The Authors

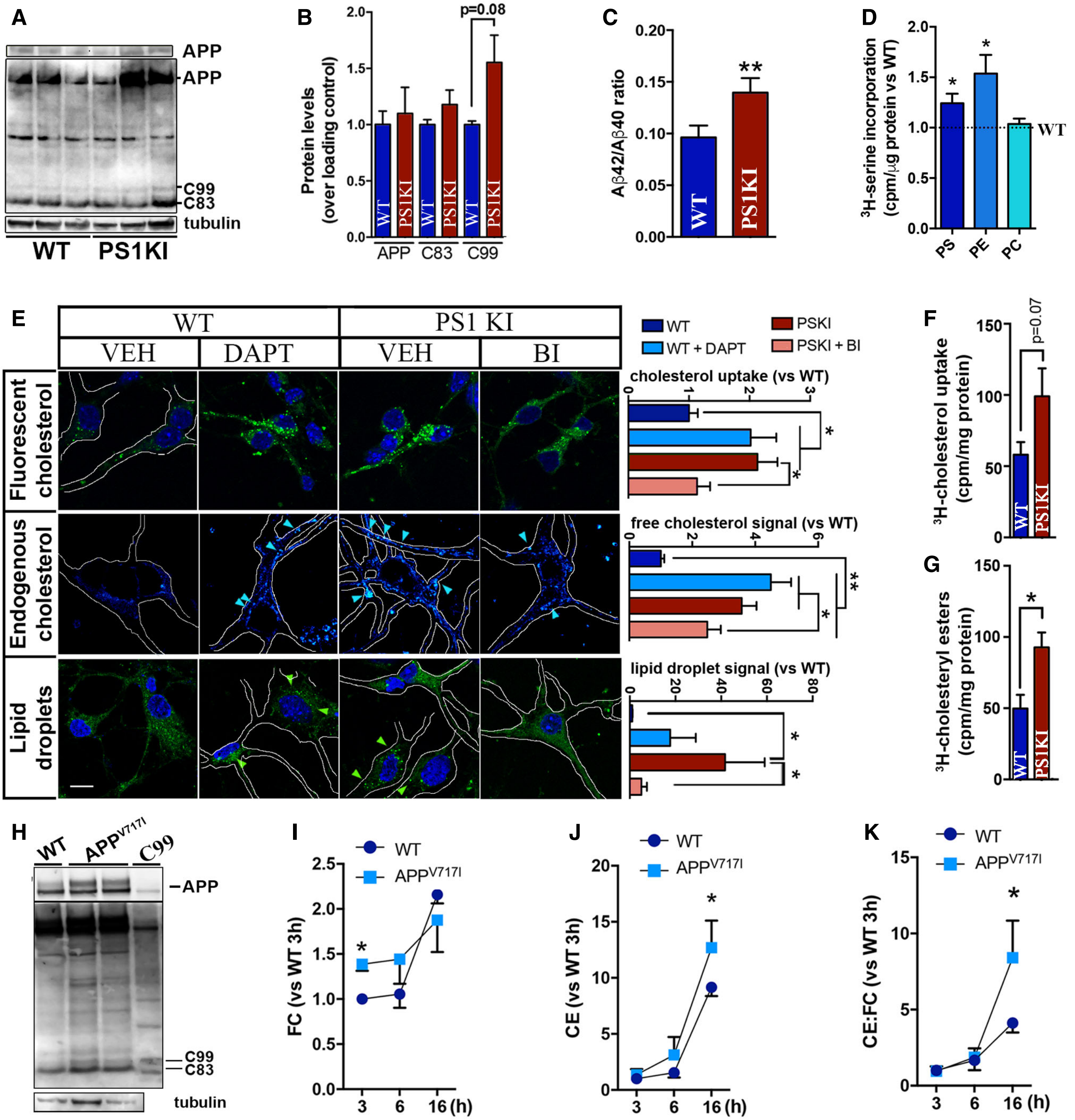
A
APP
APP
C99
C83
tubulin
WT
PS1KI
B
Protein levels (over loading control)
p=0.08
APP
C83
C99
C
Aβ42/Aβ40 ratio
**
WT
PS1KI
D
³H-serine incorporation (cpm/μg protein vs WT)
PS
PE
PC
WT
E
WT
PS1 KI
VEH
DAPT
VEH
BI
Fluorescent cholesterol
Endogenous cholesterol
Lipid droplets
WT
WT + DAPT
PSKI
PSKI + BI
cholesterol uptake (vs WT)
free cholesterol signal (vs WT)
lipid droplet signal (vs WT)
F
³H-cholesterol uptake (cpm/mg protein)
p=0.07
G
³H-cholesteryl esters (cpm/mg protein)
H
WT
APPV717I
C99
APP
C99
C83
tubulin
I
FC (vs WT 3h)
J
CE (vs WT 3h)
K
CE:FC (vs WT 3h)
(h)

Figure EV2.

Figure EV2. Neurons from PS1KI mice present with increased levels of MAM-localized C99 and elevations in cholesterol turnover.

A Western blot showing the levels of APP, C99, and C83 in homogenates of cortical neurons explanted from WT and $PS1KI^{M146V}$ mice. Upper panel shows a shorter exposure of the blot to reveal unsaturated signal of APP. Tubulin-β was used as a loading control.

B, C Graphs showing the quantification for each APP fragment (B) and the ratio $A\beta_{42}$ and $A\beta_{40}$ levels. Unpaired *t*-test (n = 6; P = 0.08) (C) in WT and $PS1KI^{M146V}$ cortical neurons. Unpaired *t*-test (n = 6; $^{**}P < 0.01$).

D Quantification of MAM activity by measurement of phospholipid synthesis and transfer between ER and mitochondria in WT and PS1KI cultured cortical neurons (Montesinos *et al*, 2020). Graphs represent the levels of ^{3}H-serine incorporation into phosphatidylserine (PS), and its subsequent conversion into phosphatidylethanolamine (PE) and phosphatidylcholine (PC) during the indicated times. Dashed line represents control levels. One-sample *t*-test (n = 6; $^{*}P < 0.05$).

E Representative confocal images of cultured cortical neurons from WT or PS1-KI mice treated with DAPT or BI, as indicated. Cholesterol uptake was analyzed by incubation with NBD-cholesterol as before (upper panel, nuclei in blue was stained by DAPI). Endogenous cholesterol (no exogenous cholesterol added) was stained with filipin (middle panels), and cholesterol esterification and subsequent lipid droplet quantification (no exogenous cholesterol added) was visualized by staining with Lipidtox (lower panel, nuclei in blue). Graphs on the right represent the level of fluorescence intensity quantified by ImageJ. Scale bar = 20 μm. Arrows indicate the presence of filipin punctae or lipid droplets. One-way ANOVA (30–50 cells/condition from 5 images from at least 3 independent experiments; post hoc: $^{*}P < 0.05$, $^{**}P < 0.01$).

F, G Quantification of (F) cholesterol uptake and (G) esterification by assessing the radioactivity levels in total homogenates from cultured cortical neurons from WT and PS1-KI mice after 4 h incubation with 2.5 μCi/ml ^{3}H-cholesterol for 4 h. Unpaired *t*-test (n = 6 for WT, n = 5 for PS1KI; $^{*}P < 0.05$).

H Western blot analysis of homogenates from APP^{V717I} cells and isogenic controls to reveal APP, C99 and C83 levels. Total homogenate of C99-transfected APP-DKO cells were used as a control for C99 signal. Tubulin-β is shown as a loading control.

I, J Quantification of cholesterol uptake and esterification in APP^{V717I} and isogenic control cell lines measured by incubation with 2.5 μCi/ml ^{3}H-cholesterol and tracking of (I) its internalization (FC, free cholesterol) Two-way repeated measures ANOVA (Time 3 and 6 h, Mutation): Mutation: $F_{(1,3)}$ = 14.36, $P < 0.05$, η = 0.36), and (J) incorporation into ^{3}H-cholesteryl esters (CE) at the indicated time points. Two-way repeated measures ANOVA (Time, Mutation) (n = 4; $^{*}P < 0.05$).

K Ratio of cholesteryl esters (CE):free cholesterol levels (CE:FC) measured by lipidomics analysis. Two-way repeated measures ANOVA (Time, Mutation) (n = 4; $^{*}P < 0.05$).

Source data are available online for this figure.

© 2020 The Authors

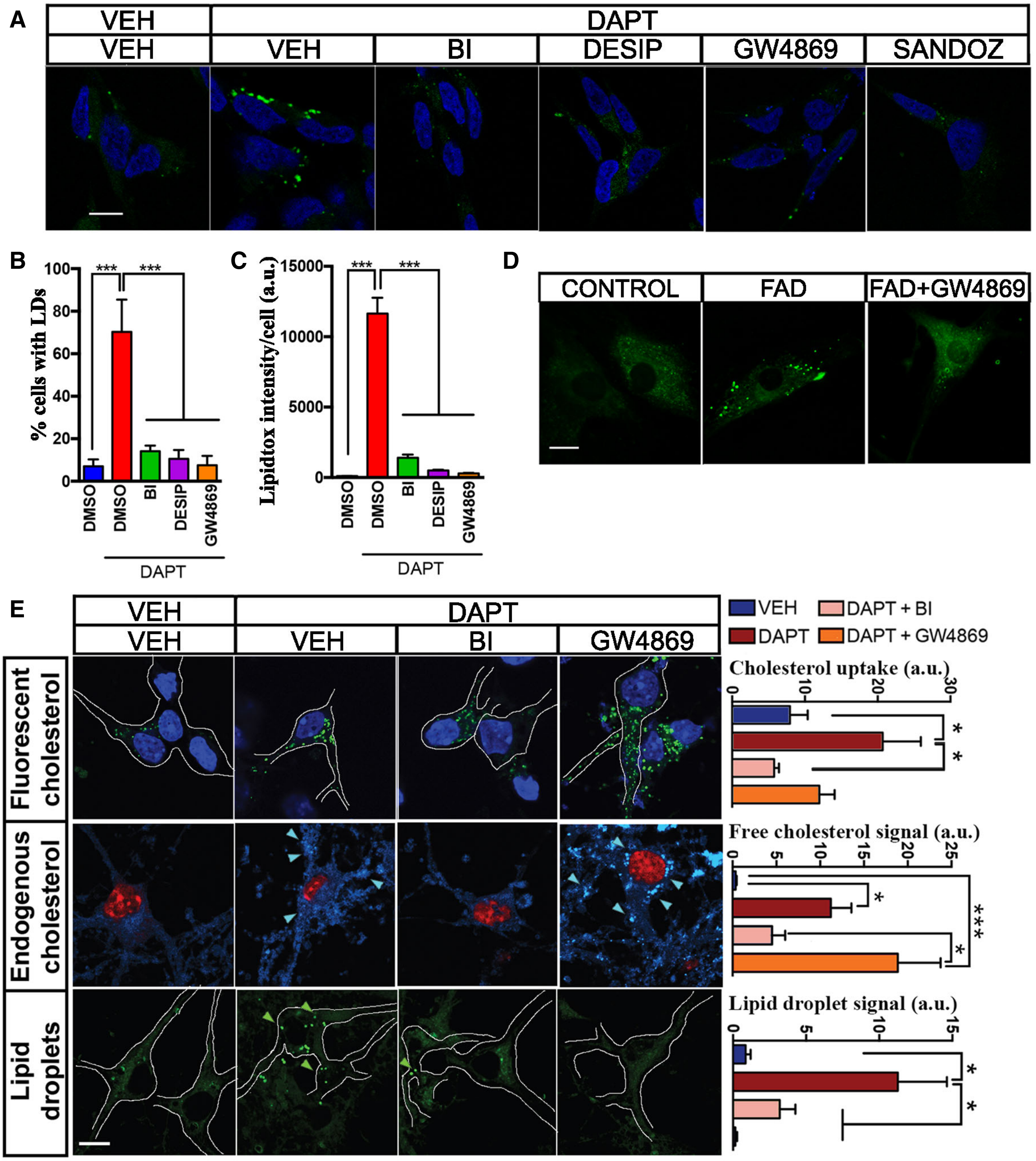
A
VEH
DAPT
VEH
VEH
BI
DESIP
GW4869
SANDOZ
B
% cells with LDs
***
***
DMSO
DMSO
BI
DESIP
GW4869
DAPT
C
Lipidtox intensity/cell (a.u.)
***
***
DMSO
DMSO
BI
DESIP
GW4869
DAPT
D
CONTROL
FAD
FAD+GW4869
E
VEH
DAPT
VEH
VEH
BI
GW4869
Fluorescent cholesterol
Endogenous cholesterol
Lipid droplets
VEH
DAPT
DAPT + BI
DAPT + GW4869
Cholesterol uptake (a.u.)
Free cholesterol signal (a.u.)
Lipid droplet signal (a.u.)

Figure EV3.

Figure EV3. Upregulation of cholesterol uptake induced by elevated C99 results in the activation of sphingomyelinase activity.

A Confocal images of human neuroblastoma cells (SH-5YSY) incubated with the indicated treatments for 12–16 h or DMSO (VEH) before staining with LipidTox to visualize lipid droplets (green). Treatment with Sandoz 58-035, a specific ACAT1 inhibitor, caused a ~95% reduction in cholesterol esterification. Nuclei are shown in blue (DAPI). Scale bar = 20 μm. One-way ANOVA (n = 5; ***$P < 0.001$).

B, C Graphs showing (B) the percentage of cells containing lipid droplets (LDs) and (C) the quantification of fluorescent intensity/cell (ImageJ). Note how elevations in cholesterol esterification can be induced by DAPT and rescued by incubation with either 100 nM BACE inhibitor (BI) or the SMase inhibitors, desipramine (DESIP, 10 μM), or GW4869 (5 μM). For Lipidtox intensity/cell. One-way ANOVA (n = 5; ***$P < 0.001$).

D Confocal images of control and FAD fibroblasts incubated with SMase inhibitor, GW4869, for 12–16 h and stained with LipidTox to detect lipid droplet formation. Scale bar = 20 μm.

E Representative confocal images of cortical neurons from WT mice, incubated with the indicated inhibitors. Cholesterol uptake was assessed by internalization of fluorescent cholesterol analog (NBD-cholesterol 2 μM; upper panel, nuclei in blue); endogenous free cholesterol levels are shown by filipin staining (middle panel, nuclei in red); and cholesterol esterification and lipid droplet formation were revealed by LipidTox staining (lower panel). Arrows indicate the presence of filipin punctae or lipid droplets. Graphs on the right represent fluorescence intensity measured by ImageJ. Scale bar = 20 μm. a.u., arbitrary units. One-way ANOVA with Greenhouse-Geisser correction. (30–80 cells/condition from 3 independent experiments; post hoc, *$P < 0.05$, ***$P < 0.001$).

Source data are available online for this figure.

 © 2020 The Authors

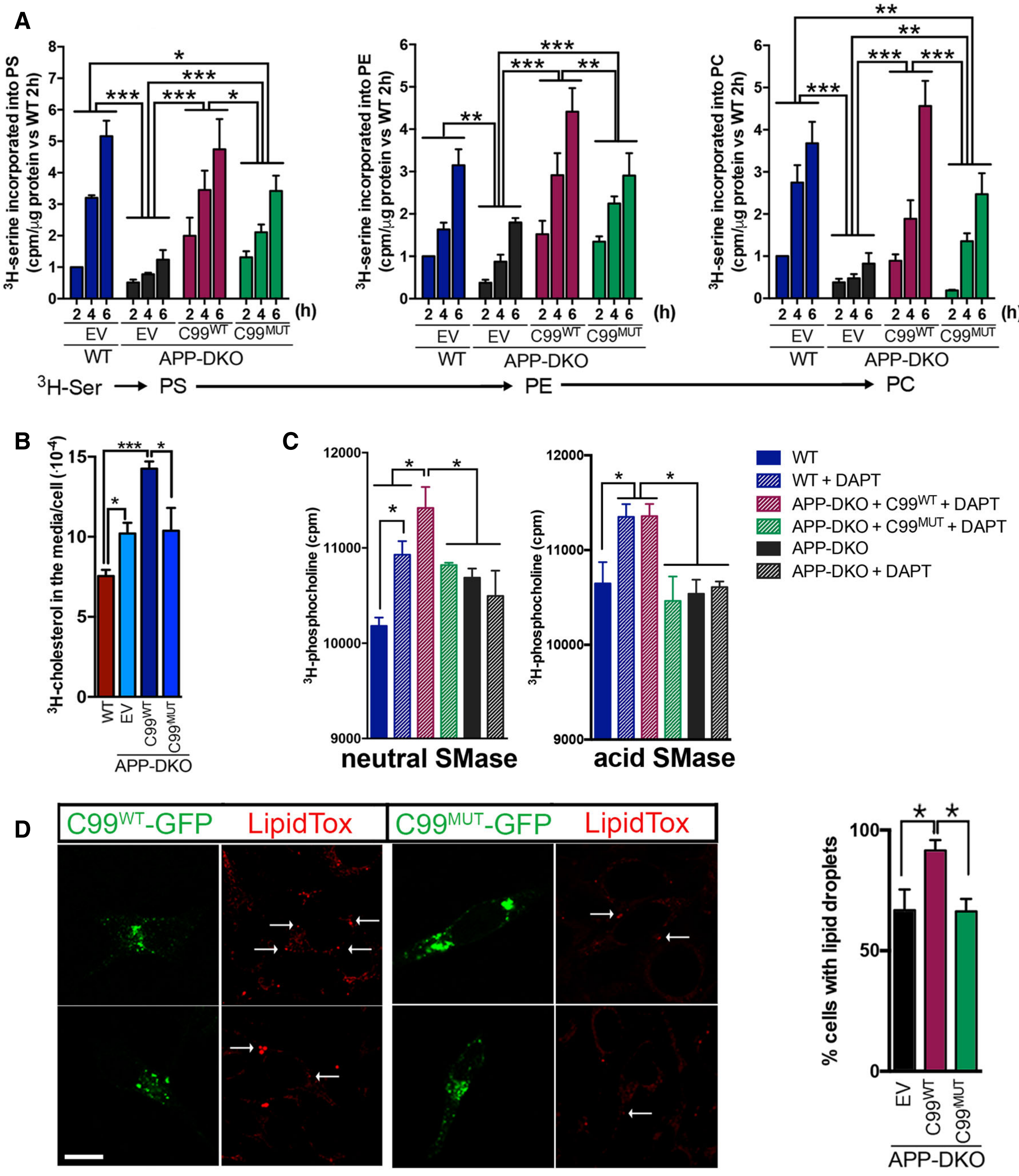

Figure EV4. C99's cholesterol-binding domain facilitates MAM formation and activation.

A Quantification of MAM activity by measurement of phospholipid synthesis and transfer between ER and mitochondria in the indicated cells (Montesinos *et al*, 2020). Graphs represent the levels of ^{3}H-serine incorporation into phosphatidylserine (PS) and its subsequent conversion into phosphatidylethanolamine (PE) and phosphatidylcholine (PC) over the indicated time periods. Two-way ANOVA (Time, Group) (n = 3; *$P < 0.05$, **$P < 0.01$, ***$P < 0.001$).

B Cholesterol efflux, measured 4 h after completion of 1 h pulse-chase with ^{3}H-cholesterol was assayed in APP-DKO or WT (APP-WT) cells transfected with an empty vector (EV) or the C99^{WT} or C99^{MUT} constructs. One-way ANOVA (n = 3; *$P < 0.05$, ***$P < 0.001$).

C Quantification of neutral and acidic SMase activities in the indicated conditions. One-way ANOVA (n = 3; *$P < 0.05$).

D Representative images of APP-DKO cells expressing C99^{WT}-GFP or C99^{MUT}-GFP and stained with LipidTox (in red) to detect lipid droplets (white arrows). Scale bar = 15 μm. Graph on the right shows the proportion of cells with lipid droplets. EV, empty vector. One-way ANOVA (n = 3; *$P < 0.05$).

Source data are available online for this figure.

© 2020 The Authors
