# Supplementary material for: The Alzheimer's disease‐associated C99 fragment of APP regulates cellular cholesterol trafficking
Source: EMBO J. 2020 Aug 31;39(20):e103791. doi: 10.15252/embj.2019103791 (PMC7560219; doi:10.15252/embj.2019103791)
Supplement: Supplementary file 3 — Source Data for Expanded View and Appendix [file EMBJ-39-e103791-s008.zip › Appendix_and_EV_Source_Data/Source_Data_Figure_EV4.pdf]

## EV4A 3H-serine incorporated (cpm/ug protein vs WT 2h)

| PS | WT | | | APP DKO | | | C99WT | | | C99 MUT | | |
|---|---|---|---|---|---|---|---|---|---|---|---|---|
| 2h | 1 | 1 | 1 | 0.5572783 | 0.3335993 | 0.6464257 | 2.174632 | 0.9204772 | 2.89696 | 1.421875 | 0.9343936 | 1.584459 |
| 4h | 3.057353 | 3.339189 | 3.205877 | 0.8667032 | 0.7569572 | 0.7026507 | 3.474265 | 2.385686 | 4.501126 | 1.881893 | 1.843936 | 2.601351 |
| 6h | 5.820466 | 4.197635 | 5.457456 | 1.669181 | 1.39588 | 0.6619361 | 6.339461 | 3.027833 | 4.871622 | 3.704779 | 2.48509 | 4.079392 |

| PE | WT | | | APP DKO | | | C99WT | | | C99 MUT | | |
|---|---|---|---|---|---|---|---|---|---|---|---|---|
| 2h | 1 | 1 | 1 | 0.464 | 0.427299 | 0.2353163 | 2.146226 | 1.306667 | 1.111546 | 1.572524 | 1.135714 | 1.325832 |
| 4h | 1.655782 | 1.354487 | 1.89759 | 1.130612 | 0.9281766 | 0.5598355 | 3.896226 | 2.728291 | 2.112352 | 2.096698 | 2.579365 | 2.059687 |
| 6h | 3.880952 | 2.937867 | 2.628389 | 2.005291 | 1.720558 | 1.672494 | 5.33805 | 3.422857 | 4.485323 | 3.79717 | 1.977778 | 2.946836 |

| PC | WT | | | APP DKO | | | C99WT | | | C99 MUT | | |
|---|---|---|---|---|---|---|---|---|---|---|---|---|
| 2h | 1 | 1 | 1 | 0.3574683 | 0.2465109 | 0.5326087 | 0.6042771 | 0.9496623 | 1.118455 | 0.1855602 | 0.1549197 | 0.2158926 |
| 4h | 3.529677 | 2.132534 | 2.580746 | 0.5813446 | 0.2821602 | 0.5638587 | 1.002502 | 2.291125 | 2.359466 | 1.028983 | 1.689603 | 1.337921 |
| 6h | 4.56813 | 2.810845 | 3.66087 | 1.320862 | 0.6247833 | 0.5176631 | 3.544977 | 4.526737 | 5.611195 | 1.576837 | 3.292354 | 2.538418 |

## EV4B 3H-cholesterol in media/cell

| APP WT | | | APP DKO EV | | | APP DKO C99WT | | | $APP^{DKO}$ C99MUT | | | |
|---|---|---|---|---|---|---|---|---|---|---|---|---|
| 0.0007992 | 0.0007326 | 0.0007326 | 0.00094391 | 0.00106734 | 0.0010503 | 0.00146483 | 0.00143582 | 0.00137781 | 0.00095489 | 0.00120301 | 0.00095489 | |

## EV4C 3H-phosphocholine (cpm)

| neutral SMase | | | | | |
|---|---|---|---|---|---|
| WT | WT DAPT | APPDKO | APP DKO DAP | c99wt | c99mut |
| 10345 | 10753 | 10592 | 10737 | 11825 | 10833 |
| 10044 | 11210 | 10783 | 9966 | 11368 | 10776 |
| 10154 | 10822 | 10454 | 10786 | 11063 | 10853 |

| acid SMase | | | | | |
|---|---|---|---|---|---|
| WT | WT DAPT | APPDKO | APP DKO DAP | c99wt | c99mut |
| 10546 | 11584 | 10753 | 10725 | 11432 | 10810 |
| 11079 | 11348 | 10612 | 10579 | 11108 | 9965 |
| 10320 | 11121 | 10253 | 10522 | 11534 | 10619 |

## EV4D % cells with lipid droplets

| APPDKO | c99wt | c99mut |
|---|---|---|
| 73.6842 | 85.7142 | 75 |
| 49.666 | 96.23475 | 57.1428 |
| 76.92 | 88.88889 | 66.6666 |
