# Supplementary material for: The Alzheimer's disease‐associated C99 fragment of APP regulates cellular cholesterol trafficking
Source: EMBO J. 2020 Aug 31;39(20):e103791. doi: 10.15252/embj.2019103791 (PMC7560219; doi:10.15252/embj.2019103791)
Supplement: Supplementary file 3 — Source Data for Expanded View and Appendix [file EMBJ-39-e103791-s008.zip › Appendix_and_EV_Source_Data/Source_Data_Figure_EV1.pdf]

EV1A

free cholesterol			Cholesterol esters		
WT	FAD	SAD	WT	FAD	SAD
97.3	158.6	154.0	87.18	343.59	197.44
105.4	154.0	148.7	55.01	408.16	358.51
98.3	155.4	165.1	141.72	328.21	166.90
99.0	163.6	146.7	116.08	209.09	145.08
		153.6			179.65
		147.4			215.87
		146.6			357.79
		151.7			197.62

EV1B HMGCR activity (% of WT)

WT			FAD		
mean	SD	n	mean	SD	n
100		3	88.7356	3.319	3

EV1D 3H-cholesterol uptake (% of control)

PS1 A246E			PS2N141I			PS1G209V			PS1M146V		
mean	SD	n	mean	SD	n	mean	SD	n	mean	SD	n
149.5652	17.39131	3	121.7391	12.17391	3	125.2174	8.695652	3	139.1304	10.43478	3

EV1E 3H-cholesterol uptake (% of WT)

WT			PS1-KD			PS1/2-KD		
mean	SD	n	mean	SD	n	mean	SD	n
100		3	335.0055	17.70492	3	432.7869	46.44809	3

EV1G 3H-cholesterol in media/cell

	wt			wt dapt			ps dko			psdko + bi		
2h	0.000717	0.000835	0.000766	0.000514	0.000931	0.001160	0.001406	0.000951	0.000951	0.000906	0.001195	0.001151
4h	0.000945	0.001274	0.001336	0.001710	0.001608	0.001329	0.001357	0.001544	0.001439	0.001262	0.001223	0.001223
6h	0.001622	0.001726	0.001934	0.002768	0.002286	0.002455	0.001999	0.002569	0.002089	0.002068	0.001540	0.001740

EV1H CE:FC

WT	FAD	SAD
0.170117	0.411855	0.2435008
0.09922	0.5036702	0.4581385
0.240372	0.4014199	0.392105
0.145344	0.242751	0.1879328
		0.2223746
		0.278381
		0.4639325
		0.2476705

Fig EV1C

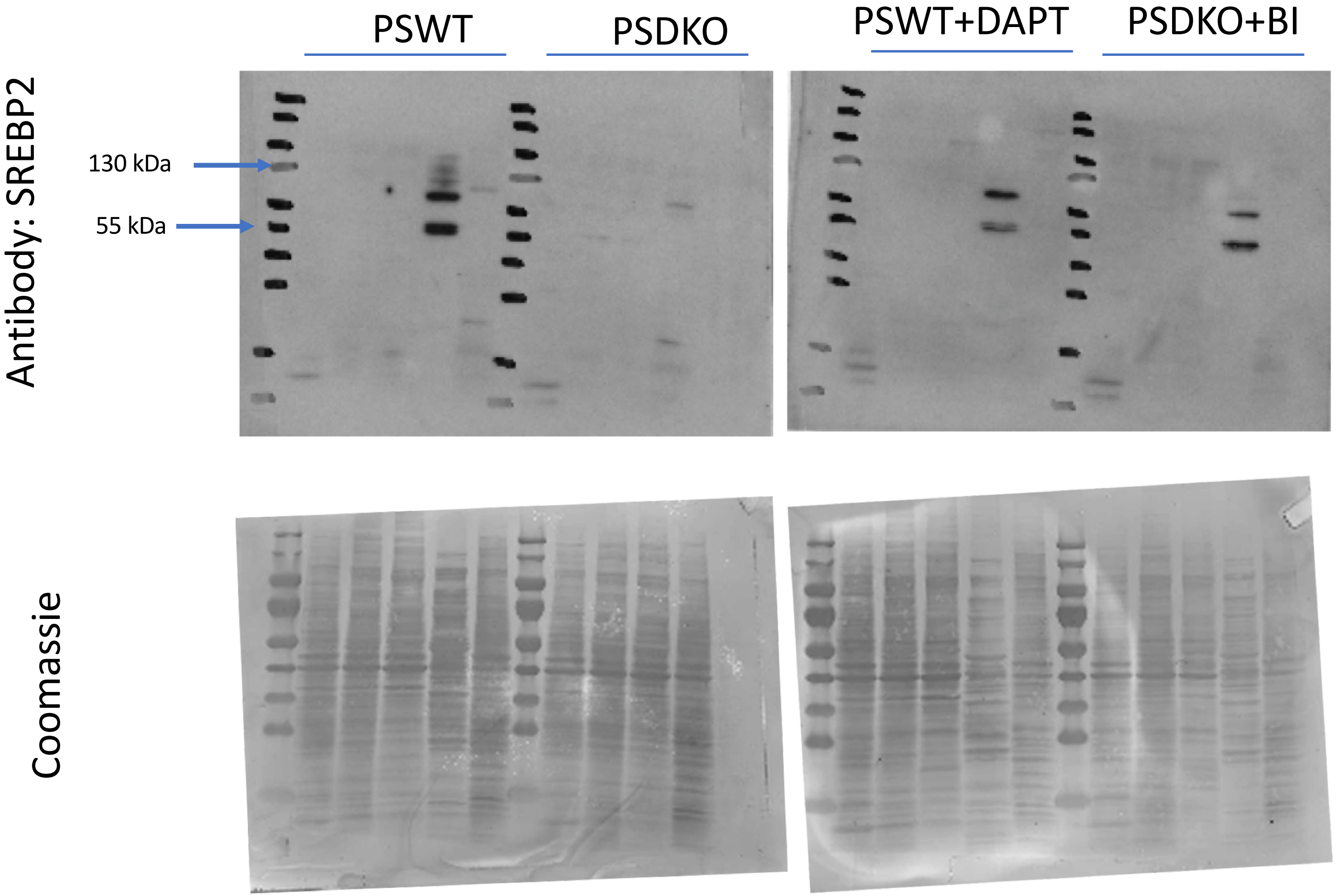

Lane order:
1.Crude membrane
2.MAM
3.MER
4.ER (used for the Figure)
5.Free mitochondria

Fig. EV1F

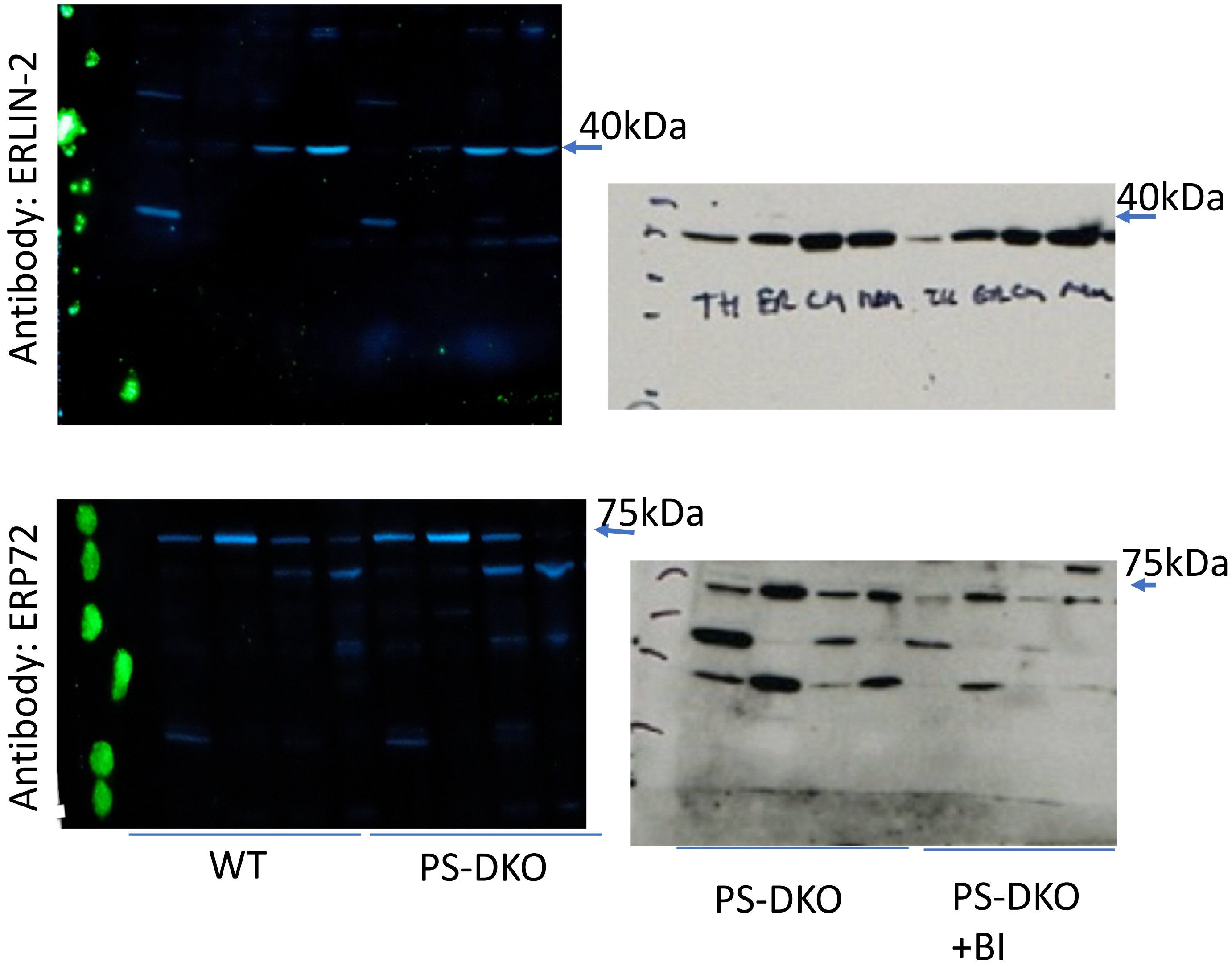

Lane order: TH/ER/CM/MAM
