# Supplementary material for: The Alzheimer's disease‐associated C99 fragment of APP regulates cellular cholesterol trafficking
Source: EMBO J. 2020 Aug 31;39(20):e103791. doi: 10.15252/embj.2019103791 (PMC7560219; doi:10.15252/embj.2019103791)

EV2B protein levels (over loading control)

	C99			C83			APP			
WT	1.033133	1.03247	0.9343967	0.91582	1.068479	1.015704	1.222721	0.9607071	0.8165715	
PS1KI	1.19621	1.446931	2.016291	1.41313	0.9660077	1.153226	0.846106	1.560936	0.8924888	

EV2C Ab42/Ab40 ratio

WT	PS1KI
0.111676	0.119718
0.09854	0.152207
0.088003	0.141357
0.086904	0.14529

EV2D 3H-serine incorporation (cpm/ug protein vs WT)

PS	PE	PC
1.041582	1.6	1.065
1.767948	1.172152	1.237864
1.299465	1.481953	0.9480122
1.24426	2.557823	1.166667
1.026275	1.118799	1.077093
1.173189	1.568182	0.8167614
1.138969	1.269231	0.9455081

EV2E cholesterol uptake (vs WT); upper panel

WT	WT + DAPT	PSKI	PSKI + BI
0.9302845	1.493339	2.937382	1.504964
0.8924754	1.581033	1.48597	0.2475099
1.065571	3.624295	1.360571	2.087863
1.454112	1.317555	1.115789	2.500426
0.6575569	1.839206	1.842869	0.1189064
2.076916	2.567433	2.81329	2.333632
0.4984807	5.674809	4.149102	2.09145
0.3373589	4.683035	3.870208	0.861501

1.972893	0.5386351	2.502151	0.3376542
0.1143516	0.689873	1.527431	0.00205731
0.9703617	0.7466902	2.954543	1.380772
1.051177	1.679475	3.924055	0.6773649
1.09963	0.7485047	0.4614739	0.6276872
0.878832	1.061884	0.182912	1.446046
		0.8293561	0.7723494

EV2E free cholesterol signal (vs WT); middle panel

WT			WT + DAPT			PSKI			PSKI + BI		
mean	SD	n	mean	SD	n	mean	SD	n	mean	SD	n
1	0.3669	5	4.498	1.119	5	3.572	1.334	5	2.479	1.051	5

EV2E lipid droplet signal (vs WT); lower panel

WT	WT + DAPT	PSKI	PSKI + BI
0.1040604	0.02081208	2.800704	0.3456284
0.7831585	2.346734	2.164169	0.6028307
0.9866737	4.663479	2.430547	0
2.278917	0.2185263	1.906529	1.170676
0.8471902	0.0260151	3.03335	0.5318629
1.312549	127.2919	36.69373	21.86071
1.953734	121.0015	140.5	27.10185
0.07040504	2.332962	108.8962	13.62334
1.584107	2.217751	107.9605	2.428968
0.07920568	0.8208562	210.7967	3.769088
1.549243	1.927147	0.3697792	0
1.684802	1.374144	6.998595	0.01446875
0.8652811	0.6323205	1.401265	0
0.4199623	0.6751095	2.011093	0.1566124
0.4807121	0.3697777	4.239235	0.1830397

EV2F 3H-cholesterol uptake (cpm/mg protein)

WT	PS1KI
61419.15	79734.07
78941.61	152430.2
87313.06	136268.7
33388.76	45941.54
46973.84	80709.4
40205.32	

EV2G 3H-cholesteryl esters (cpm/mg protein)

WT	PS1KI
86.00917	113.5734
30.41363	105.4131
32.95311	83.10249
28.66973	56.21135
69.95134	105.4131
50.69709	

EV2I free cholesterol (vs WT 3h)

	wt				APP V717I			
3h	1	1	1	1	1.199643	1.465621	1.536196	1.344998
6h	1.132481	0.838549	1.456804	0.79487	1.302749	0.912866	2.212505	1.346652
16h	2.274851	1.906642	2.106004	2.34837	2.217725	1.231872	1.349548	2.702688

EV2J cholesterol esters (vs WT 3h)

	wt				APP V717I			
3h	1	1	1	1	1.199643	1.465621	1.536196	1.344998
6h	1.132481	0.838549	1.456804	0.79487	1.302749	0.912866	2.212505	1.346652
16h	2.274851	1.906642	2.106004	2.34837	2.217725	1.231872	1.349548	2.702688

EV2K CE:free cholesterol (vs WT 3h)

	wt				APP V717I			
3h	1	1	1	1	1.199643	1.465621	1.536196	1.344998
6h	1.132481	0.838549	1.456804	0.79487	1.302749	0.912866	2.212505	1.346652
16h	2.274851	1.906642	2.106004	2.34837	2.217725	1.231872	1.349548	2.702688

Fig. EV2A

The first 6 lines are the lines shown in Fig. EV2A.
The last line (*) is a MEF cell line where C99 was overexpressed

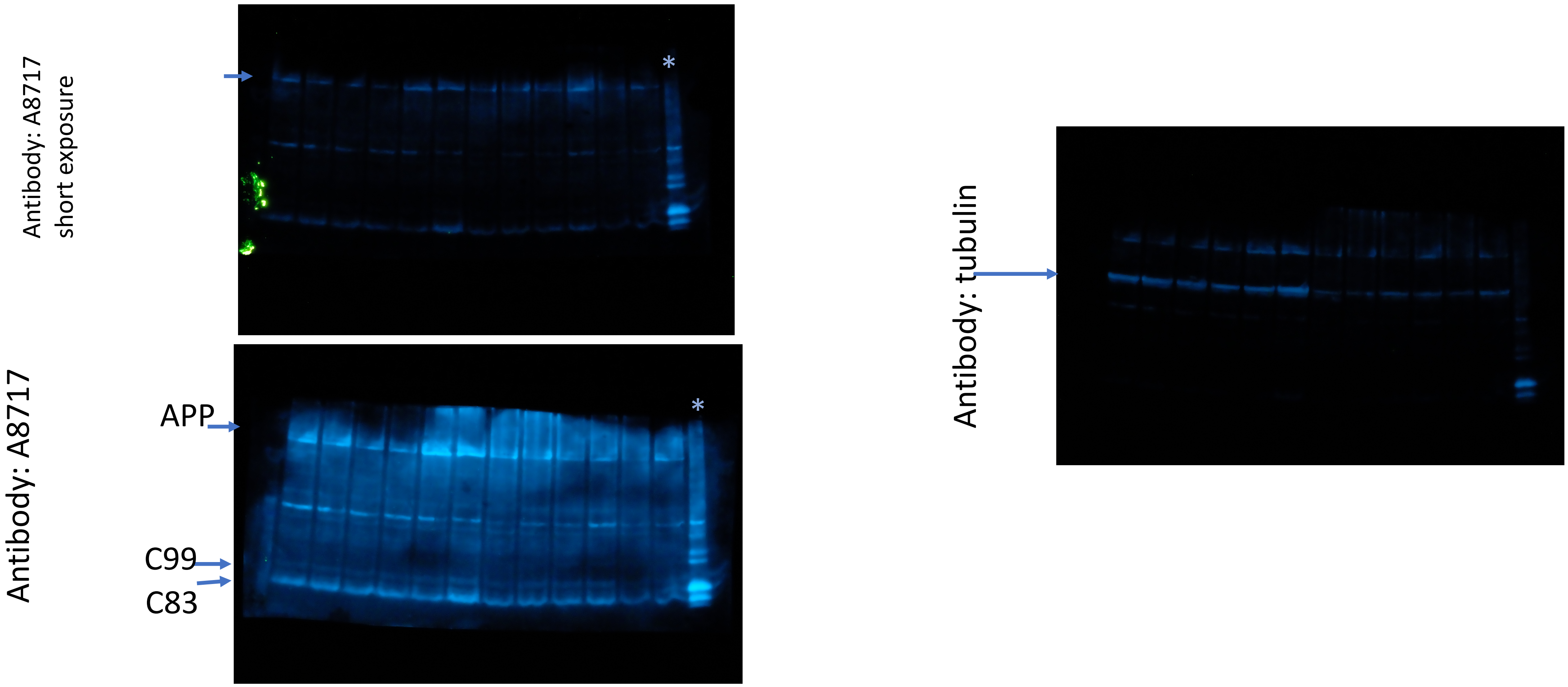

Fig. EV2H

Total homogenate from human induced pluripotent stem cells harboring the mutation V717I in APP (APPV717I, lanes 2&3) and its WT counterpart (lane 1). Also a sample where human C99 was overexpressed in a MEF cell line was loaded as a control (lane 4)

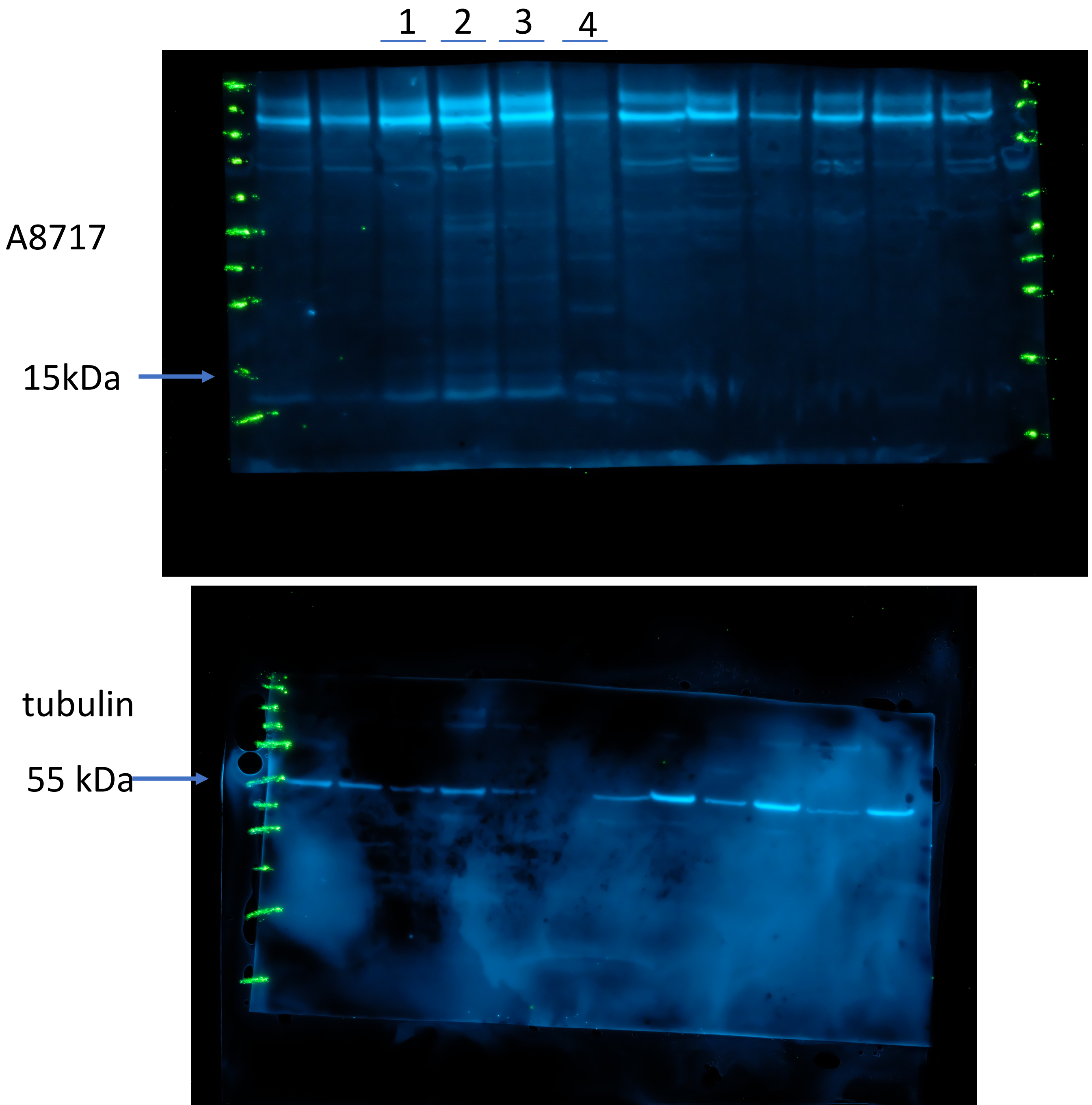

Supplement: Supplementary file 3 — Source Data for Expanded View and Appendix [file EMBJ-39-e103791-s008.zip › Appendix_and_EV_Source_Data/Source_Data_Figure_EV2.pdf]
