# Supplementary material for: The Alzheimer's disease‐associated C99 fragment of APP regulates cellular cholesterol trafficking
Source: EMBO J. 2020 Aug 31;39(20):e103791. doi: 10.15252/embj.2019103791 (PMC7560219; doi:10.15252/embj.2019103791)
Supplement: Supplementary file 3 — Source Data for Expanded View and Appendix [file EMBJ-39-e103791-s008.zip › Appendix_and_EV_Source_Data/Source_Data_Figure_EV3.pdf]

EV3B %CELLS WITH LDS

DMSO	DMSO DAPT	DAPT BI	DAPT DESIP	DAPT GW4869
6	78	16.5	5	4.5
6.5	49.5	15.5	12	8.5
3	61.5	9.5	9	1.5
12	88.5	14	10	10.5
7.5	74	15	16.5	12.5

EV3C Lipidtox intensity/cell (arbitrary units)

DMSO	DMSO DAPT	DAPT BI	DAPT DESIP	DAPT GW4869
76.60149	12631.14	1006.39	424.8032	271.6958
140.8201	8066.831	1024.309	527.0159	211.6824
121.8009	10186.79	1115.434	650.9255	283.1535
72.42559	14391.74	2062.766	571.5446	432.7921
94.91779	12970.32	1848.806	375.3114	271.442

EV3E cholesterol uptake (arbitrary units), upper panel

VEH	DAPT	DAPT + BI	DAPT + GW4869
7.2862	27.9610	7.2272	19.7678
8.3665	16.0952	6.7589	11.4049
5.8813	30.6011	6.5303	11.7994
1.5339	8.0642	4.0044	7.5332
16.5745	23.3650	4.2957	9.4233

EV3E free cholesterol signal (arbitrary units), middle panel

VEH	DAPT	DAPT + BI	DAPT + GW4869
0.3002536	17.31752	2.458155	14.31747
0.8545917	8.306315	8.95371	31.19008
0.3904877	6.793282	2.806905	21.45135
0.3482473	12.4462	3.791576	8.494982
0.4723562	10.46423	4.57845	16.42789

EV3E lipid droplet signal (arbitrary units), lower panel

VEH	DAPT	DAPT + BI	DAPT + GW4869
0.03687	5.93659	1.21682	0
0.81121	7.89088	7.04279	0.51623
0.14749	15.70203	3.24485	0
1.76992	19.0066	3.39234	0.07375
1.50741	9.24607	1.06932	0
