# Supplementary material for: The Alzheimer's disease‐associated C99 fragment of APP regulates cellular cholesterol trafficking
Source: EMBO J. 2020 Aug 31;39(20):e103791. doi: 10.15252/embj.2019103791 (PMC7560219; doi:10.15252/embj.2019103791)
Supplement: Supplementary file 3 — Source Data for Expanded View and Appendix [file EMBJ-39-e103791-s008.zip › Appendix_and_EV_Source_Data/Source_Data_Appendix_Figure_1.pdf]

Appendix 1A

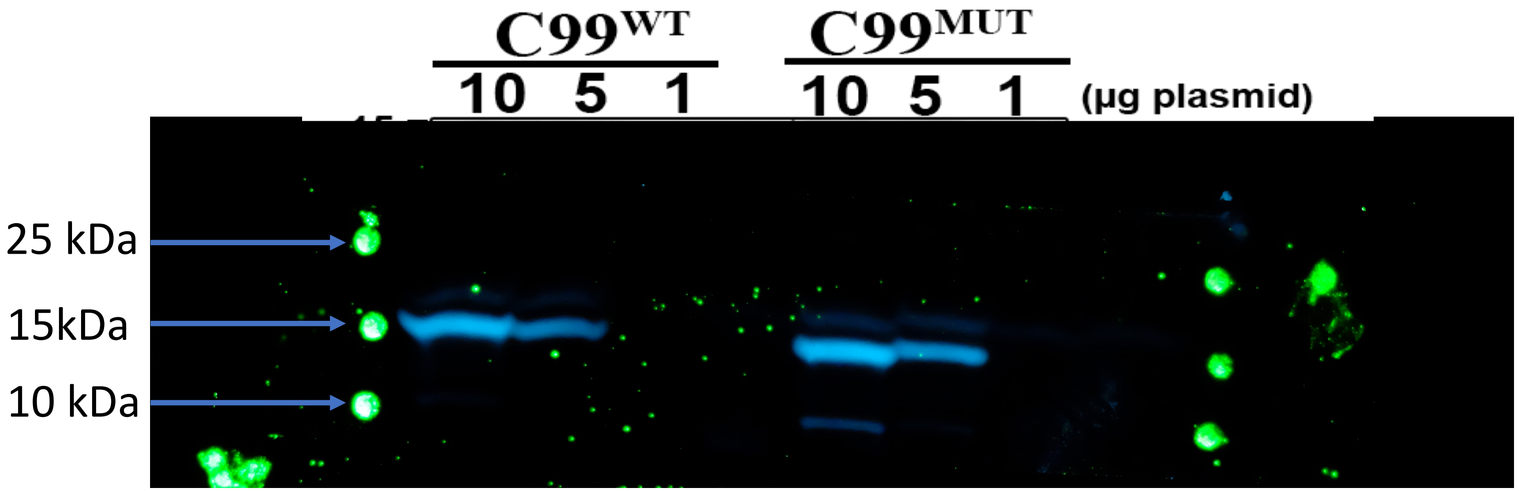

Antibody: 6E10

APP-DKO cells were transfected with different amount of plasmid expressing either C99wt or C99mut

Appendix 1B

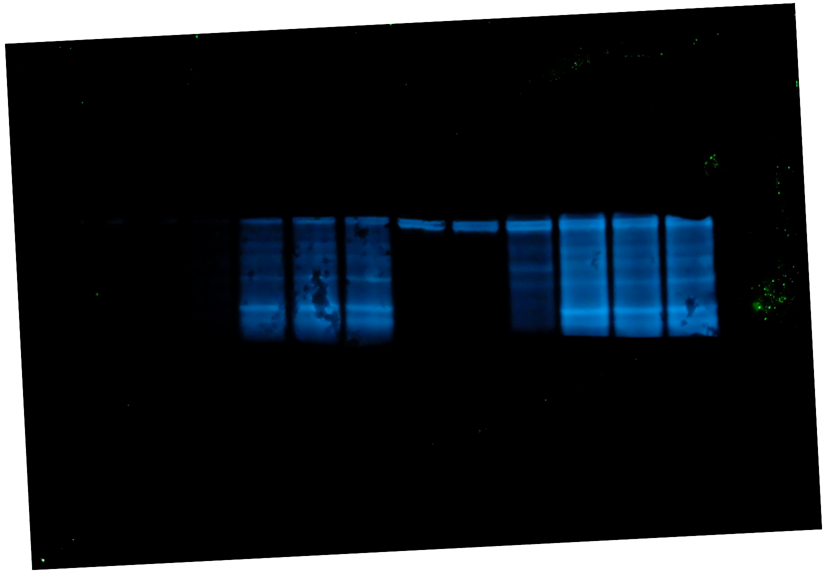

(min)	60	60	60	60	30	15	60	60	60	60	30	15
Biotin-azide	-	-	+	+	+	+	-	-	+	+	+	+
CuSO4/TBTA	-	+	-	+	+	+	-	+	-	+	+	+

Appendix 1C

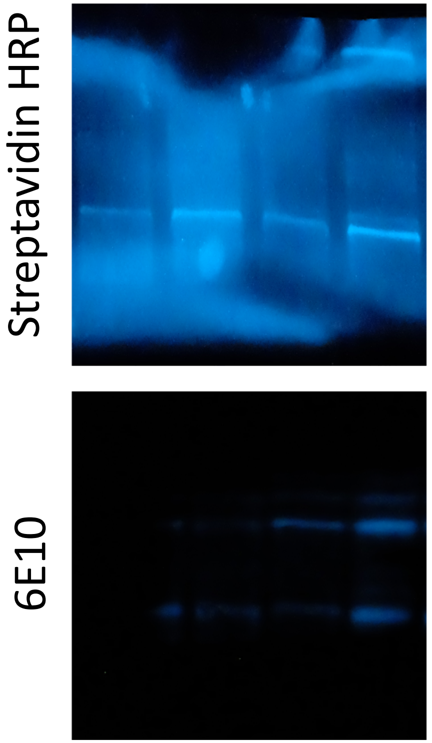

Cholesterol	-	+	+	+
UV	+	-	+	+
CLICK	+	+	-	+

Appendix 1D

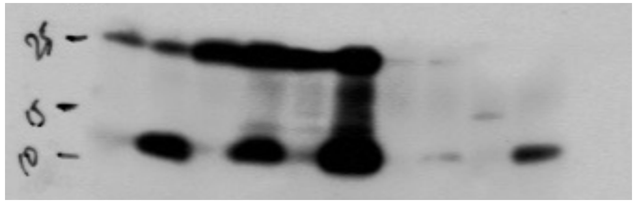

Order (from left to right)

1. TH PSwt
2. TH PSDKO
3. MAM PSwt
4. MAM PSDKO
5. Pulldown MAM PSwt
6. Pulldown MAM PSdko

Appendix 1E
(1/2)

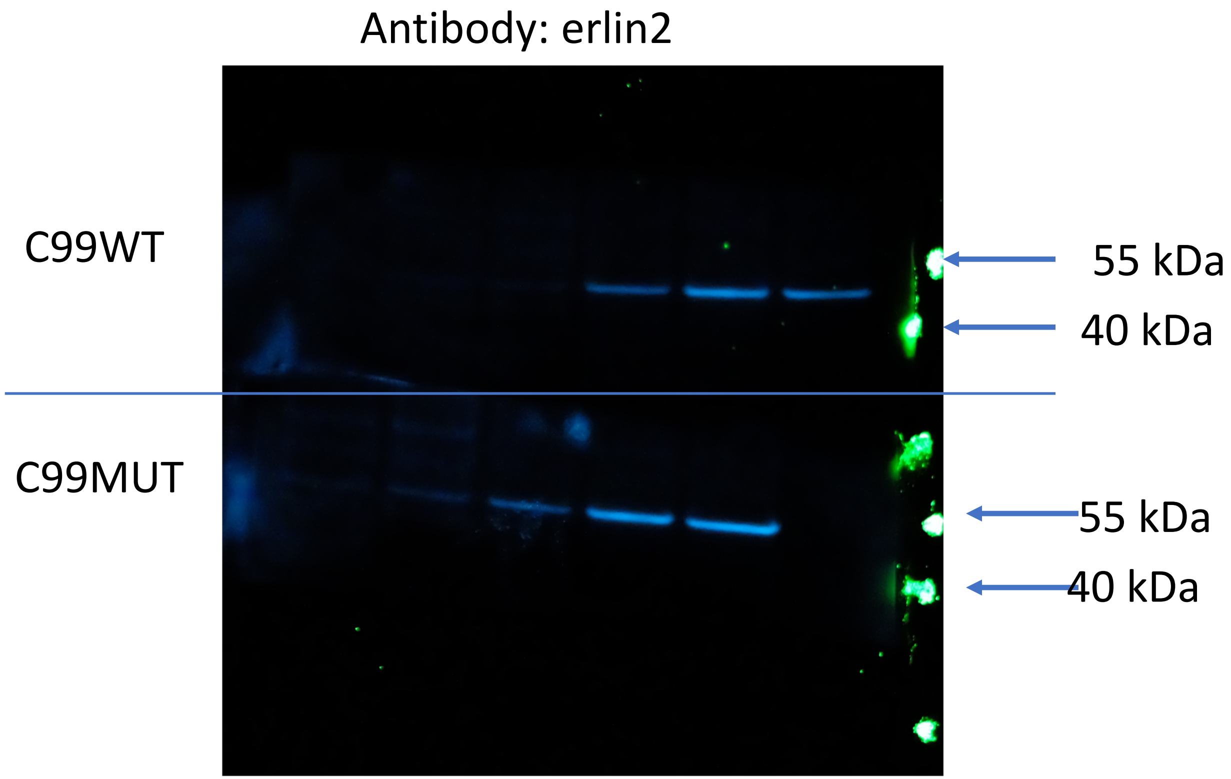

Note: Loading order TH, ER, CM, MAM, MER
Erlin-2 was run in parallel gels

Appendix 1E
(2/2)

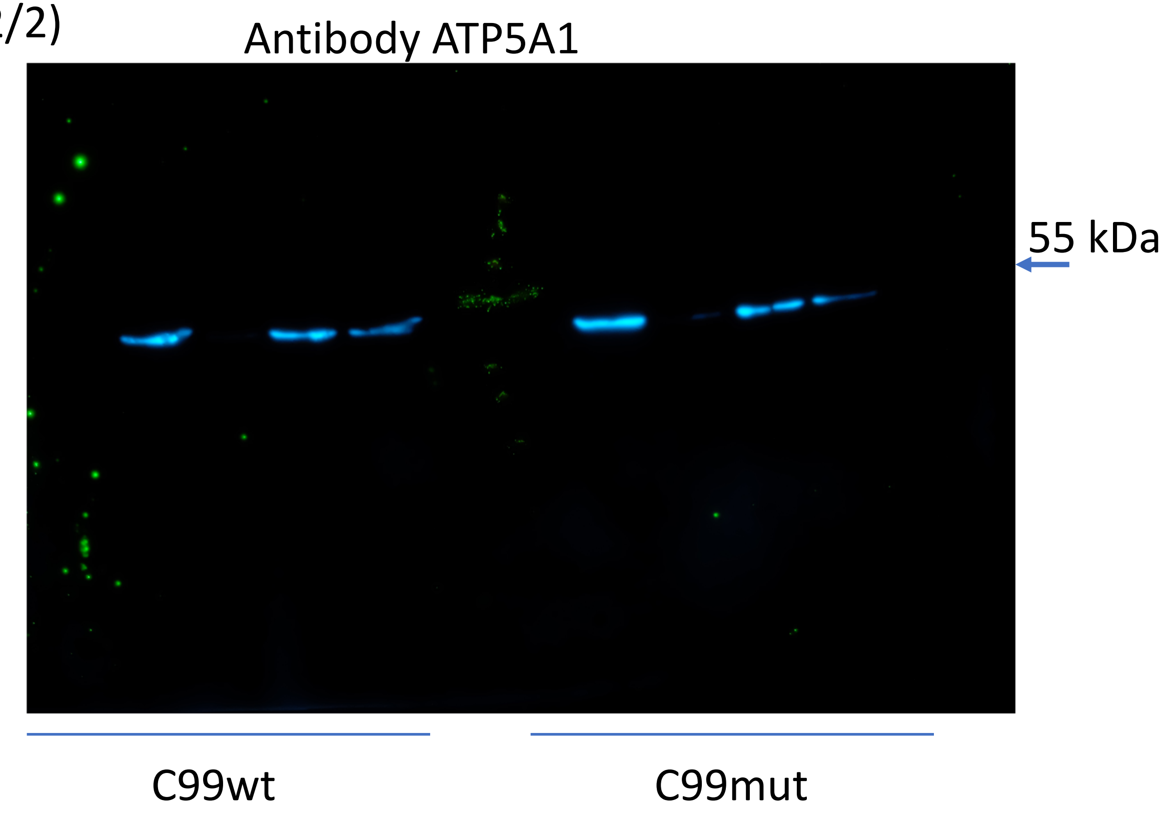

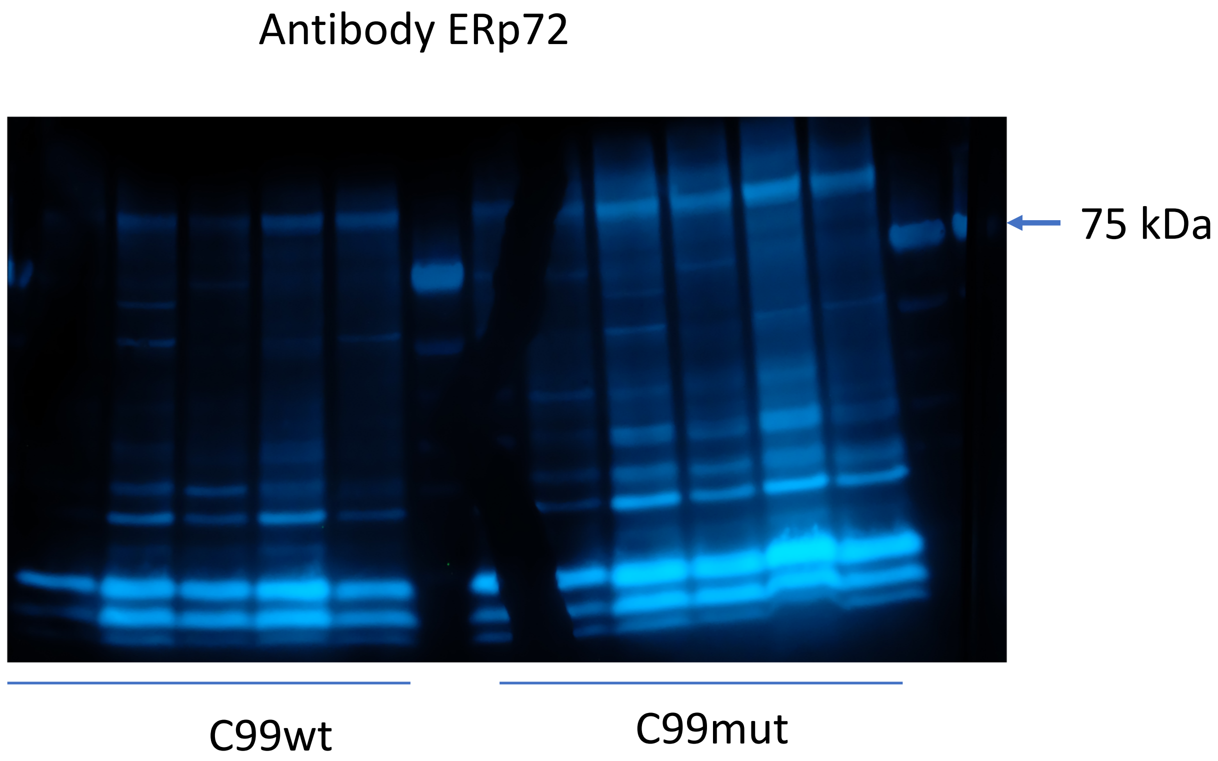

Note: Loading order TH, ER, CM, MAM, MER
