# Supplementary material for: The Alzheimer's disease‐associated C99 fragment of APP regulates cellular cholesterol trafficking
Source: EMBO J. 2020 Aug 31;39(20):e103791. doi: 10.15252/embj.2019103791 (PMC7560219; doi:10.15252/embj.2019103791)
Supplement: Supplementary file 5 — Source Data for Figure 1 [file EMBJ-39-e103791-s003.pdf]

1A Free cholesterol (% of WT)

	WT						PS-DKO						
TH	98.34	108.73	97.16	109	89.3	97.705	103	134.1	129.5	134.1	108.2	99.5	
MAM	101.36	121.6	114.2				138	165.8	180.6				
ER	63.981	82.755	85.05				87.5	71.03	68.74				

1B Free cholesterol (% of APPWT)

APP^{WT}			VEH			C99			C83			AICD			Ab		
mean	SD	n	mean	SD	n	mean	SD	n	mean	SD	n	mean	SD	n	mean	SD	n
100	5.906504	4	87.132	4.302	4	103.772	8.981	4	85.113	8.2511	4	81.17	7.6036	4	77.66	6.7614	4

1C HMGCR activity (% of WT)

WT			WT dapt			PS DKO		
mean	SD	n	mean	SD	n	mean	SD	n
100		3	80.54	1.2	3	63.473	1.38	3

1D 3H-cholesterol uptake (% of WT 2h)

	WT			Ps DKO			PS^{DKO} + BI			
2 h	100	100	100	110	142	123.66	55.9	66.67	71.43	
4 h	77.019	181.26	122.4	133	302	145.47	45.6	140.4	97.35	
6 h	82.144	195.99	136.8	150	387	176.62	67.7	158.5	119.2	

1E 3H-cholesterol internalized in MAM (% of WT 2h)

	WT			Ps DKO			PS^{DKO} + BI			
2	100	100	100	166	117	121.67	136	76.71	64.49	
4	83.378	127.56	106.3	157	147	126.17	73.9	84.08	68.43	
6	91.652	100.39	76.75	227	202	194.4	141	98.22	65.48	

1F 3H-cholesterol internalized in ER (% of WT 2h)

	WT			Ps DKO			PS^{DKO} + BI			
2	100	100	100	89	123	92.852	72.3	76.63	65.76	
4	41.662	87.447	173.8	95.6	54.2	186.85	81.8	56.58	99.99	
6	45.049	85.886	181.6	70	85.4	186.34	72.3	71.05	166.1	

1G cholesterol uptake (% of WT)

WT			WT + DAPT			PS-DKO			PS-DKO + BI		
mean	SD	n	mean	SD	n	mean	SD	n	mean	SD	n
100	0	3	273.2	49.3	3	332.24	28.4	3	130.7	53.78	3

1G cells with cholesterol puncta (%)

WT			WT + DAPT			PS-DKO			PS-DKO + BI		
mean	SD	n	mean	SD	n	mean	SD	n	mean	SD	n
12.85714	4.2857	3	42.86	5.71	3	62.857	14.3	3	28.57	10	3
