# Supplementary material for: The Alzheimer's disease‐associated C99 fragment of APP regulates cellular cholesterol trafficking
Source: EMBO J. 2020 Aug 31;39(20):e103791. doi: 10.15252/embj.2019103791 (PMC7560219; doi:10.15252/embj.2019103791)
Supplement: Supplementary file 6 — Source Data for Figure 2 [file EMBJ-39-e103791-s004.pdf]

2A fluorescence intensity/cell (arbitrary units)

WT			PS-DKO			PS-DKO + DESIP			PS-DKO + GW486[illegible]		
mean	SD	n	mean	SD	n	mean	SD	n	mean	SD	n
725.4	144.7	3	2347.8	122.6	3	1084	196.6	3	737.5	46.5	3

2B fluorescence intensity (% of VEH)

VEH			DAPT			DAPT + GW4869			DAPT + DESIP		
mean	SD	n	mean	SD	n	mean	SD	n	mean	SD	n
100	54.77	7	543.4	151.7	5	2305	291.4	5	1568	459.3	6

2C fluorescence intensity (% of VEH)

VEH			GW4869			DESIP		
mean	SD	n	mean	SD	n	mean	SD	n
100	54.77	6	350.8	51.91	6	279.5	61.34	6
