# Supplementary material for: The Alzheimer's disease‐associated C99 fragment of APP regulates cellular cholesterol trafficking
Source: EMBO J. 2020 Aug 31;39(20):e103791. doi: 10.15252/embj.2019103791 (PMC7560219; doi:10.15252/embj.2019103791)
Supplement: Supplementary file 7 — Source Data for Figure 3 [file EMBJ-39-e103791-s005.pdf]

	Profiles for 3B,3D,3F					
**fraction**	**C99 wt**	**Rab5**	**ACSL4**	**Flotillin-1**	**ATP5A1**	**SEC61B**
**1**	0.000	0.000	0.014	0.064	0.010	0.000
**2**	0.198	0.850	0.043	0.000	0.010	0.000
**3**	1.000	1.000	0.030	0.275	0.010	0.000
**4**	0.833	0.999	0.033	0.576	0.010	0.000
**5**	0.702	0.669	0.030	1.000	0.010	0.000
**6**	0.492	0.632	0.009	0.896	0.010	0.000
**7**	0.407	0.616	0.000	0.911	0.010	0.000
**8**	0.123	0.456	0.005	0.686	0.010	0.000
**8**	0.046	0.320	0.018	0.555	0.010	0.000
**10**	0.128	0.137	0.047	0.487	0.051	0.000
**11**	0.162	0.011	0.132	0.422	0.051	0.000
**12**	0.139	0.011	0.188	0.335	0.062	0.000
**13**	0.056	0.011	0.204	0.350	0.000	0.353
**14**	0.182	0.011	0.494	0.550	0.000	0.961
**15**	0.483	0.011	0.566	0.280	0.000	1.000
**16**	0.740	0.011	0.746	0.063	0.026	0.929
**17**	0.814	0.011	1.000	0.426	0.319	0.581
**18**	0.697	0.011	0.936	0.643	0.668	0.360
**19**	0.522	0.011	0.915	0.526	1.000	0.148
**20**	0.574	0.011	0.872	0.562	0.990	0.141
**21**	0.434	0.011	0.746	0.462	0.290	0.000
**22**	0.252	0.011	0.660	0.470	0.148	0.000
**23**	0.160	0.011	0.732	0.330	0.083	0.000
**24**	0.337	0.011	0.550	0.527	0.083	0.000

Profiles for 3C,3E,3G					
**C99 mut**	**rab5**	**ACSL4**	**Flotillin-1**	**ATP5A1**	**SEC61B**
1.000	0.000	0.000	0.471	0.000	0.000
0.649	0.408	0.000	0.655	0.000	0.000
0.735	0.950	0.000	1.000	0.000	0.000
0.646	1.000	0.002	0.981	0.000	0.000
0.374	0.663	0.028	0.867	0.000	0.000
0.341	0.449	0.064	0.782	0.000	0.000
0.700	0.375	0.059	0.611	0.000	0.000
0.662	0.297	0.182	0.531	0.000	0.000
0.523	0.298	0.130	0.370	0.000	0.000
0.417	0.424	0.180	0.313	0.000	0.000
0.193	0.253	0.198	0.245	0.000	0.000
0.156	0.369	0.244	0.219	0.000	0.000
0.266	0.000	0.125	0.064	0.000	0.480
0.348	0.000	0.559	0.160	0.000	0.708
0.249	0.000	0.571	0.176	0.000	1.000
0.371	0.000	0.664	0.069	0.197	0.947
0.473	0.000	0.860	0.033	0.754	0.516
0.524	0.000	0.965	0.069	0.960	0.546
0.304	0.000	0.998	0.117	1.000	0.258
0.461	0.000	0.962	0.061	0.777	0.258
0.228	0.000	1.000	0.000	0.881	0.258
0.006	0.000	0.938	0.015	0.323	0.000
0.000	0.000	0.935	0.061	0.251	0.000
0.005	0.000	0.800	0.053	0.317	0.000

Figure 3A
(1/4)

lower part of the membrane: A8717 (C99) +upper part of the membrane: ACSL4

Antibody: A8717 (C99)

Antibody: ACLS4

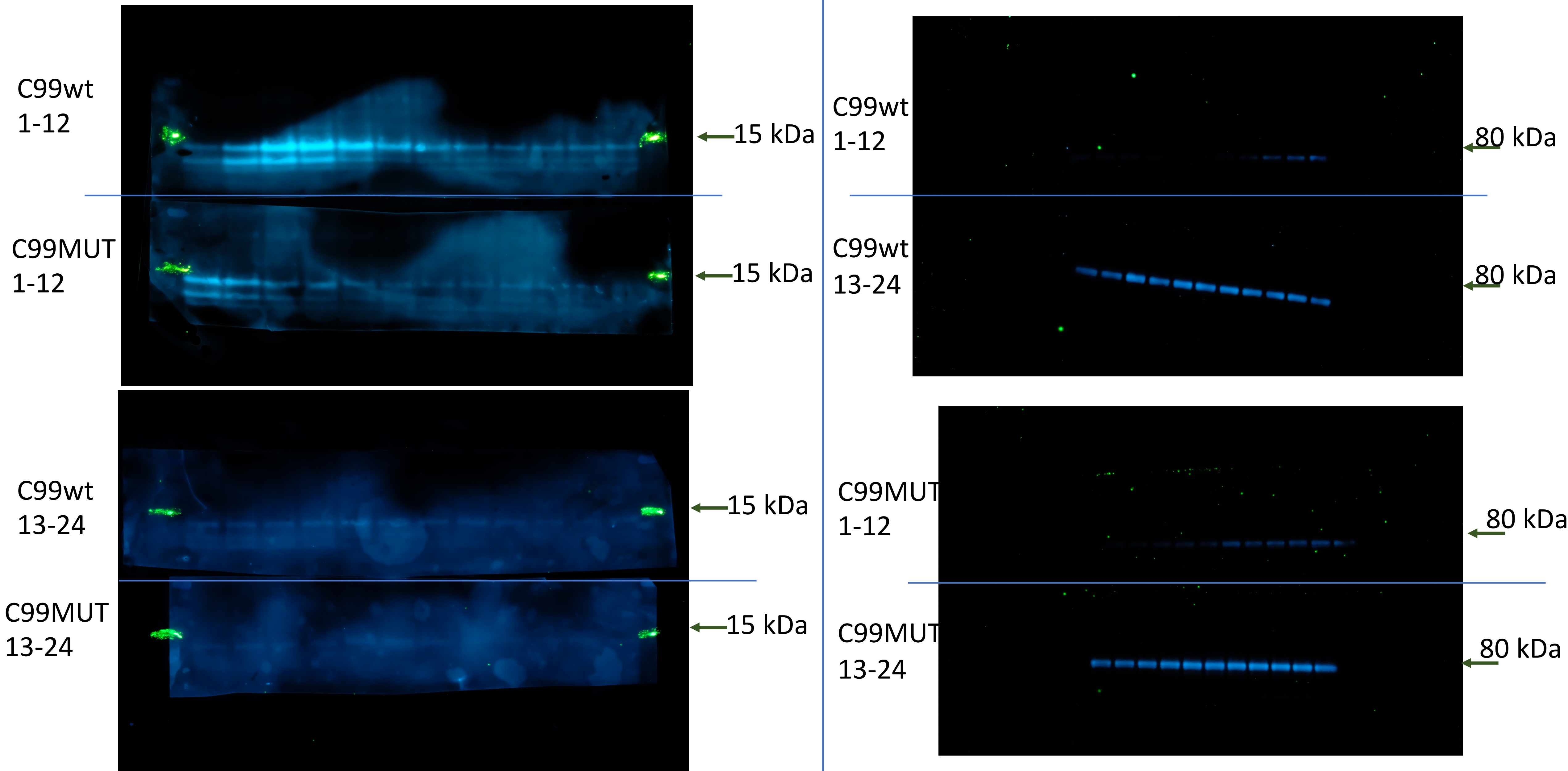

Figure 3A
(2/4)

Antibody: rab5 (immunoblotted after A8717 antibody incubation)

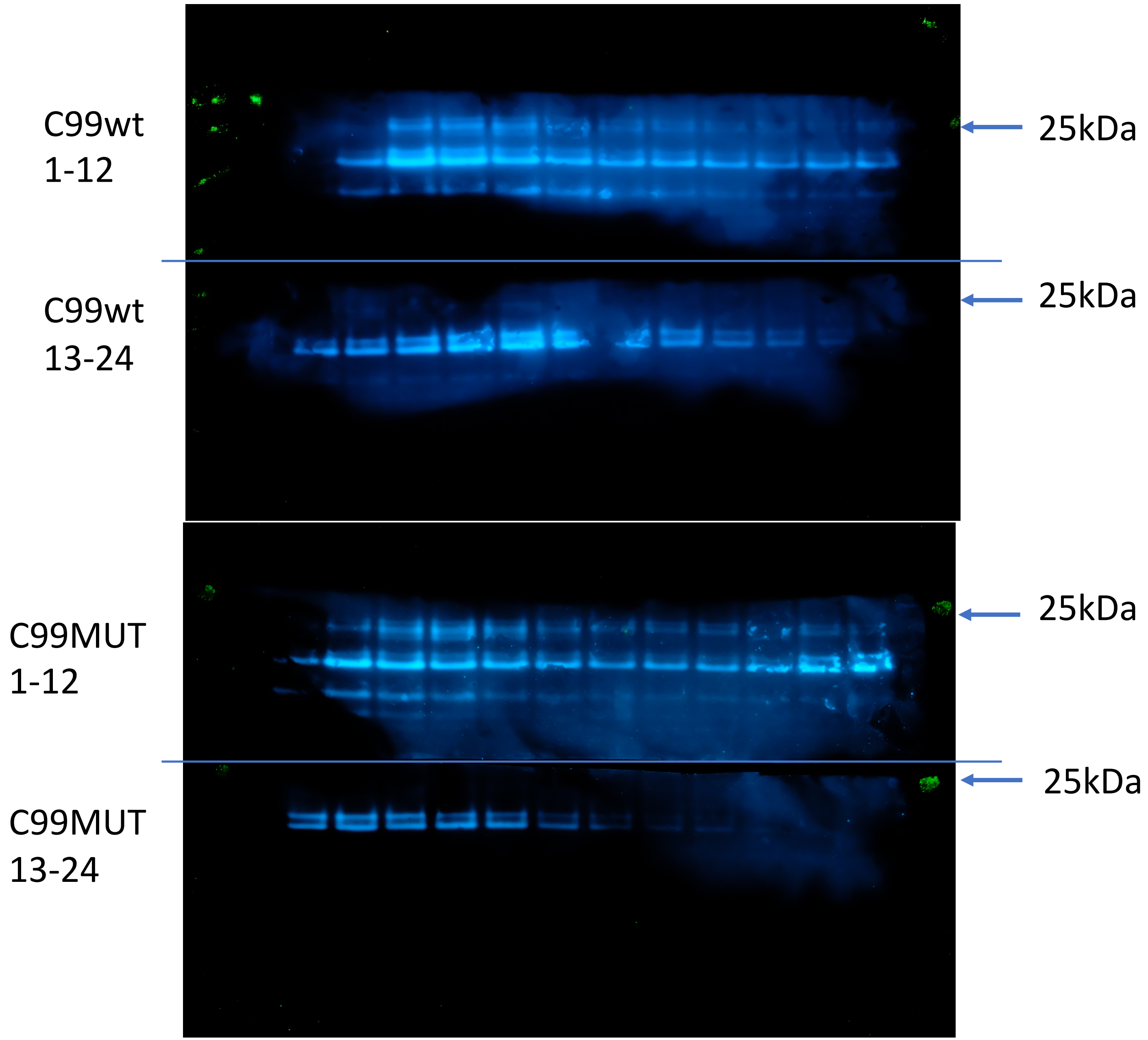

Figure 3A
(3/4)

Antibody: flotillin-1

C99wt
1-12

C99wt
13-24

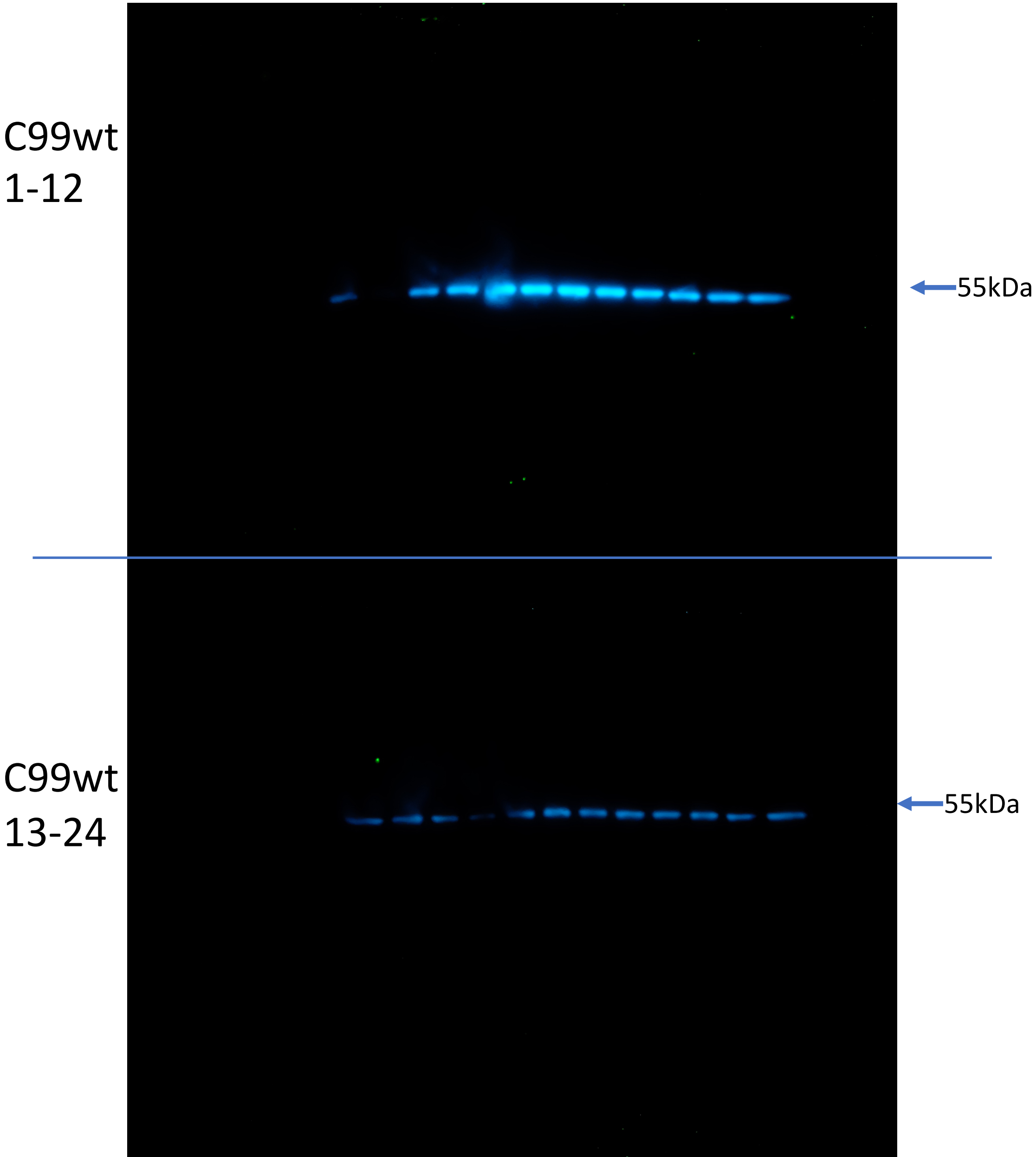

55kDa

55kDa

C99MUT
1-12

C99MUT
13-24

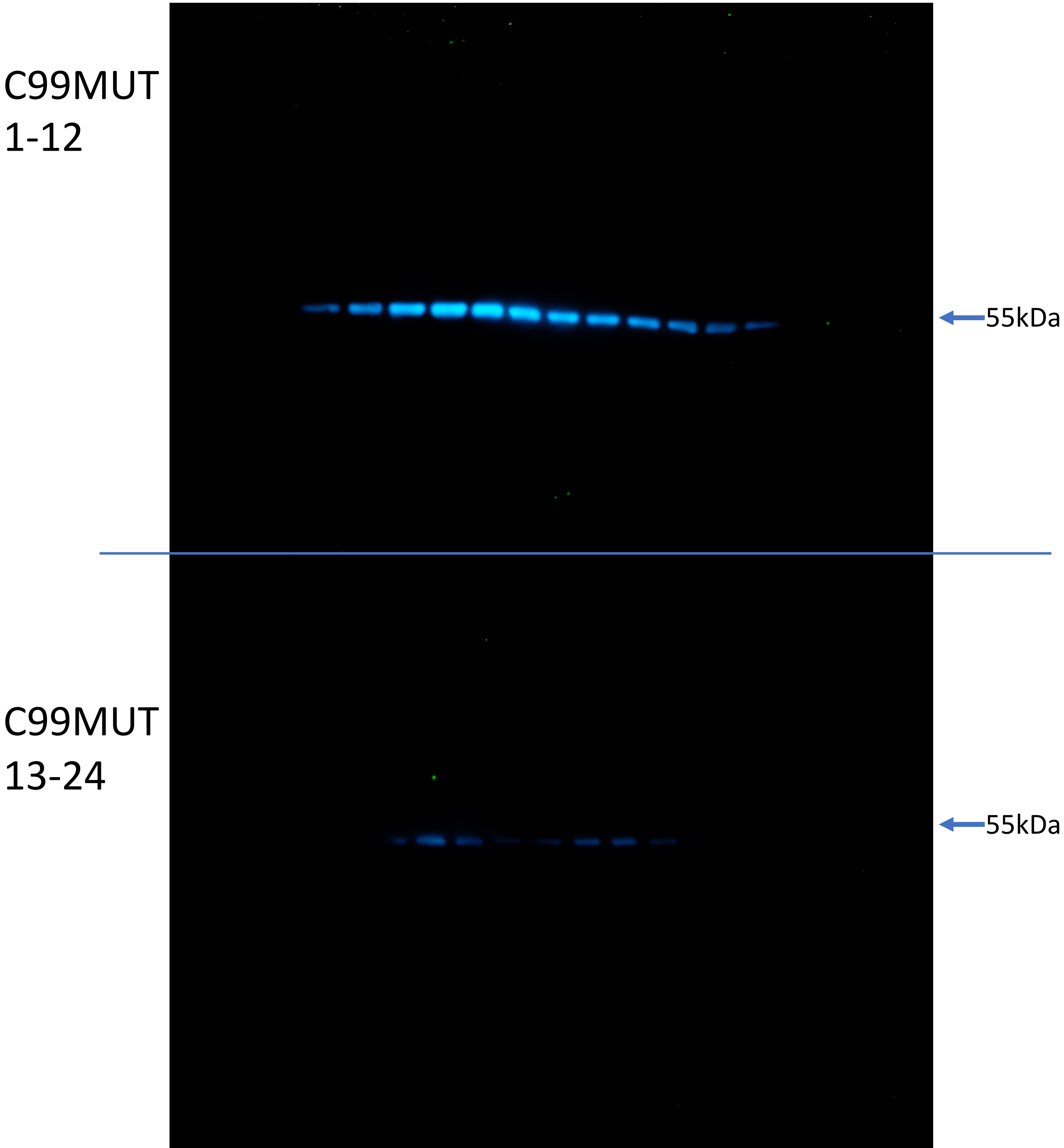

55kDa

55kDa

Figure 3A (4/4)

Antibody: ATP5A1

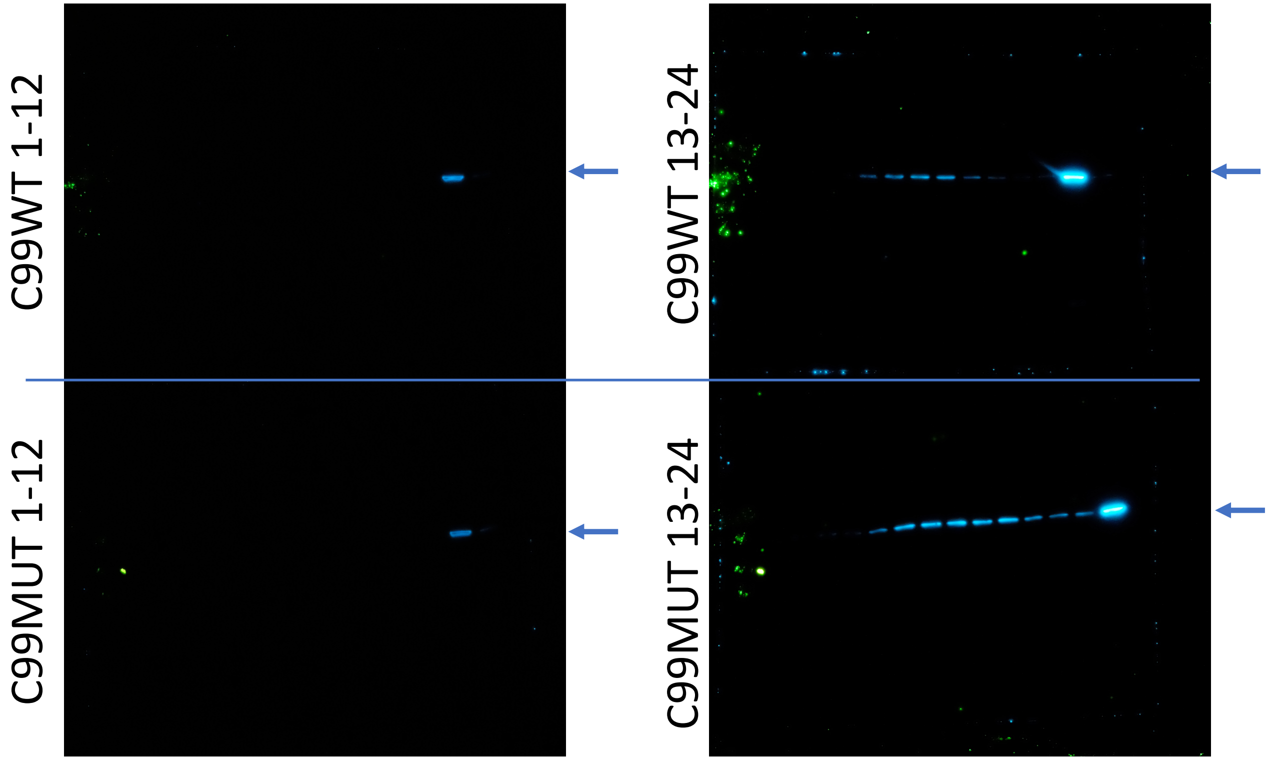

Note: arrow marks 55kDa
A total homogenate sample was loaded after the sucrose gradient fractions in each gel to control that the antibody worked properly

Antibody: SEC61B

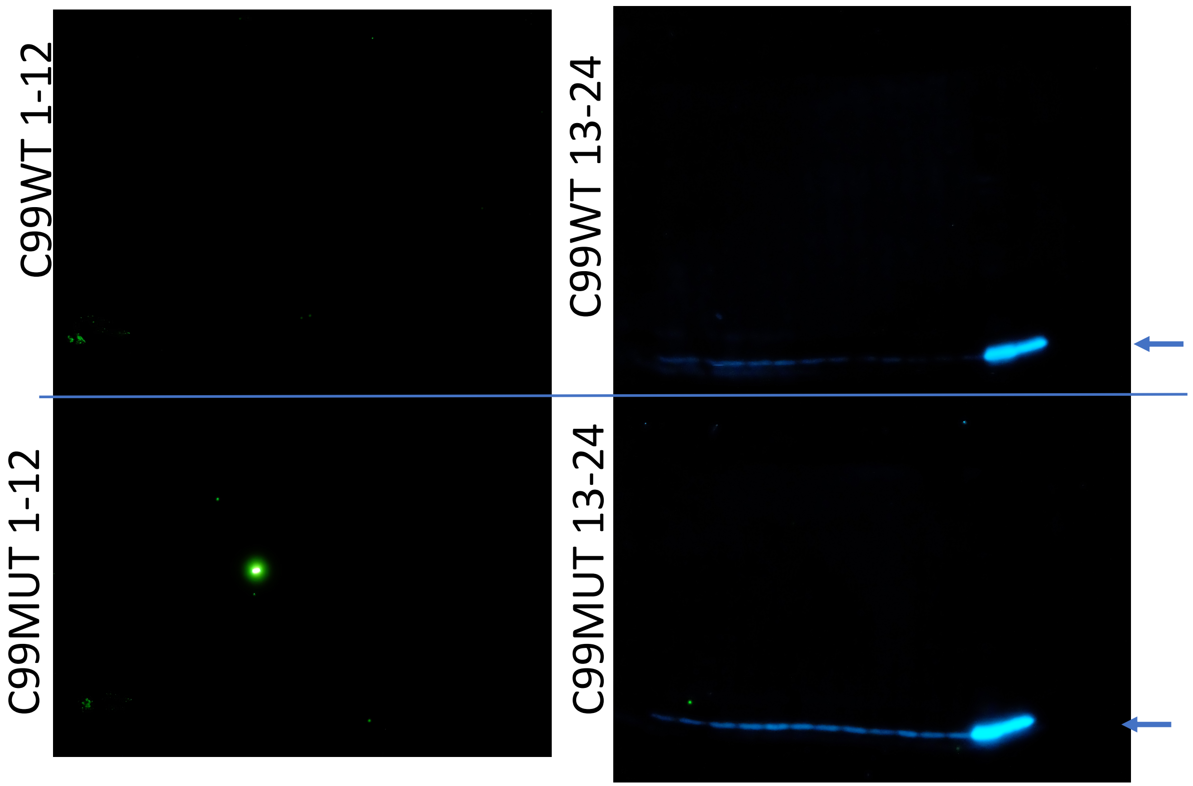

Note: arrow marks 15kDa
A total homogenate and an ER sample were loaded after the sucrose gradient fractions in the second gel to control that the antibody worked properly
