# Supplementary material for: The Alzheimer's disease‐associated C99 fragment of APP regulates cellular cholesterol trafficking
Source: EMBO J. 2020 Aug 31;39(20):e103791. doi: 10.15252/embj.2019103791 (PMC7560219; doi:10.15252/embj.2019103791)
Supplement: Supplementary file 8 — Source Data for Figure 4 [file EMBJ-39-e103791-s006.pdf]

4B C99 colocalization (arbitrary units)

	C99WT			C99MUT		
	mean	SD	n	mean	SD	n
mito	0.1028	0.05779	12	0.06095	0.02923	13
ER	0.14	0.1227	13	0.1245	0.1001	15

4D % ER-mito colocalization (per cell)

GFP			C99WT-GFP			C99mut-GFP		
mean	SD	n	mean	SD	n	mean	SD	n
20.57	11.69	14	41.35	22.78	25	28.94	16.69	28
