# Supplementary material for: The Alzheimer's disease‐associated C99 fragment of APP regulates cellular cholesterol trafficking
Source: EMBO J. 2020 Aug 31;39(20):e103791. doi: 10.15252/embj.2019103791 (PMC7560219; doi:10.15252/embj.2019103791)
Supplement: Supplementary file 9 — Source Data for Figure 5 [file EMBJ-39-e103791-s007.pdf]

5C pull down C99 levels (versus input)

	c99 wt					c99 MUT				
TH	1	0.9797188	1.097515	1.02985	0.8632485	0.2628101	0.6731788	0.7368099	0.2078631	0.8115637
MAM	0.4759867	0.409084	0.4842233	0.6552585	0.5549405	0.08495148	0.0877103	0.203072	0.5553363	0.1856856

5E Free cholesterol in MAM (% over WT)

APP-DKO			C99wt			C99mut		
mean	SD	n	mean	SD	n	mean	SD	n
87.12088	5.120879	4	103.7582	12.1978	4	82.31209	12.48352	4

5F 3H-cholesterol (vs WT)

EV	C99 DAPT	99 DAPT SANDO	C99MUT DAPT	MUT DAPT SANDOZ
0.7673537	2.337304	0.631473	0.641527	0.9312582
2.06996	3.372149	1.166107	1.892212	1.132984
1.50169	1.898177	1.69125	0.4439332	1.499115
1.487945	1.702839		1.753954	

5G 3H-cholesteryl esters (vs WT)

EV	C99 DAPT	99 DAPT SANDO	MUT DAPT	UT DAPT SANDOZ
1.00938	1.591065	0.03326422	1.190161	0.04203237
1.05389	2.15147	0.06247888	1.701211	0.0723328
0.5151765	1.28204	0.00048086	0.5278735	0.00095411

5H CE:FC (vs APP-DKO +EV)

APP-DKO + EV			C99wt			C99mut		
mean	SD	n	mean	SD	n	mean	SD	n
1	0.09156903	4	1.331038	0.127243	4	1.10177	0.08620582	4

Figure 5B

Antibody: 6E10

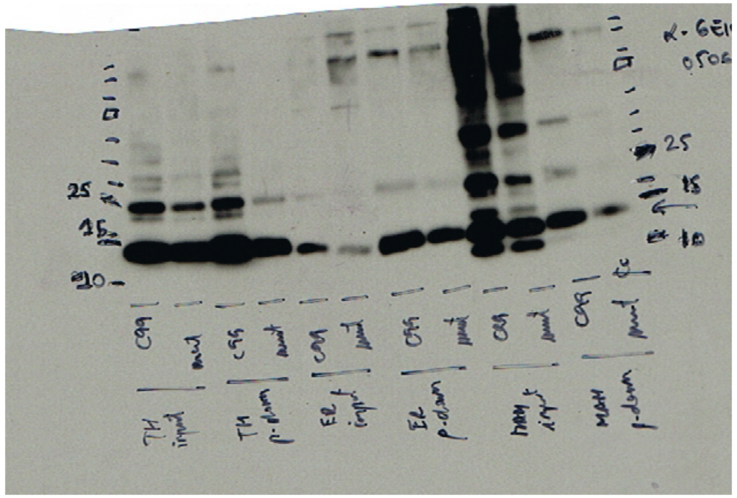

Antibody: ACLS4

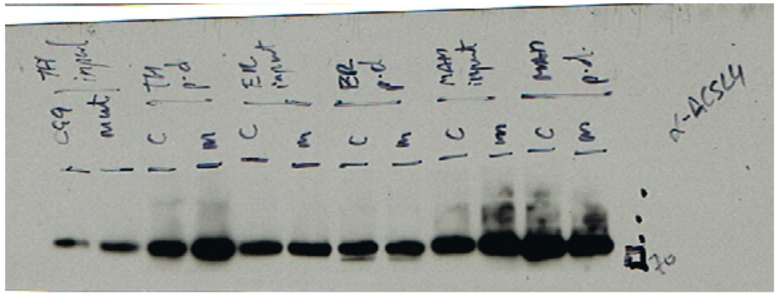

Antibody 6E10

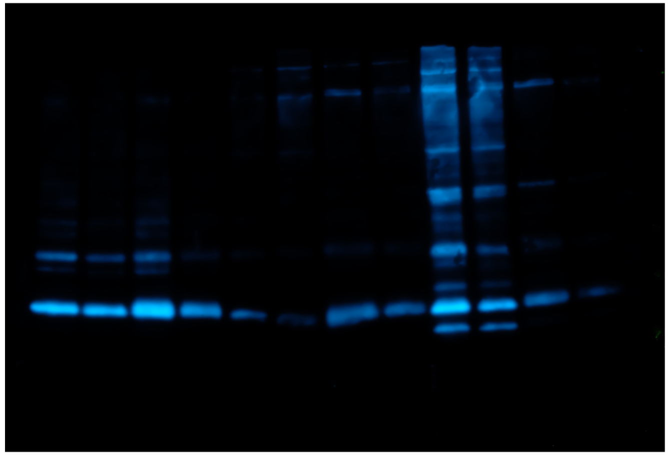

Antibody: streptavidin-HRP

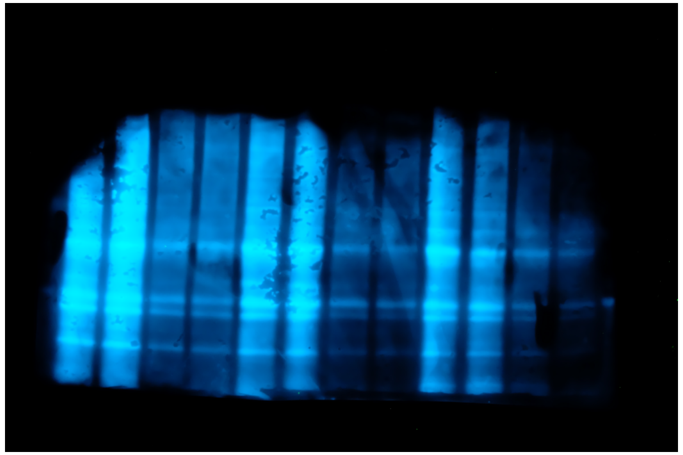

Order (from left to right)

1. TH C99wt input
2. TH C99mut input
3. Pull-down TH C99wt
4. Pull-down TH C99mut
5. ER C99wt input
6. ER C99mut input
7. Pull-down ER C99wt
8. Pull-down ER C99mut
9. MAM C99 wt input
10. MAM C99 mut input
11. Pull down MAM C99 wt
12. Pull down C99 mut

Fig. 5D

Antibody: A8717

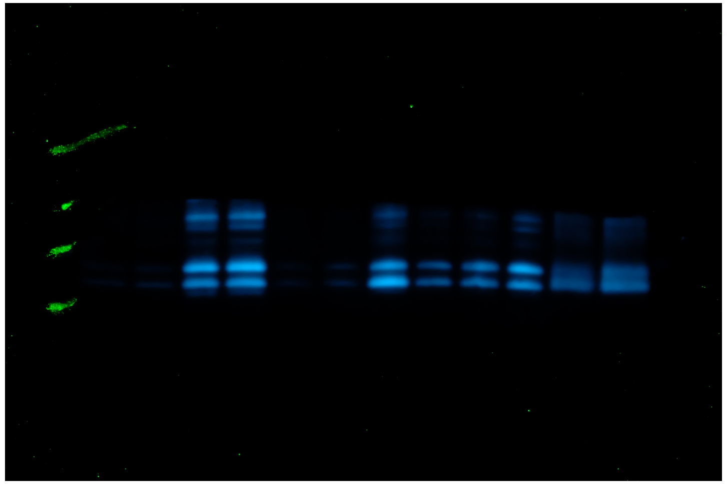

Antibody: flotilin

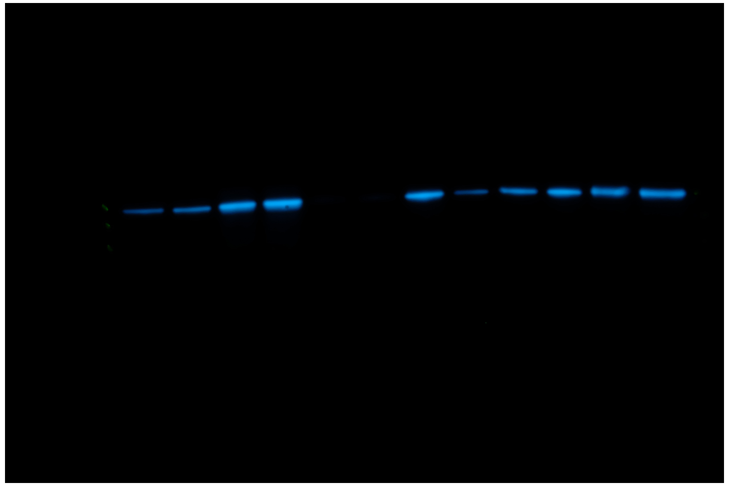

Antibody: streptavidin HRP

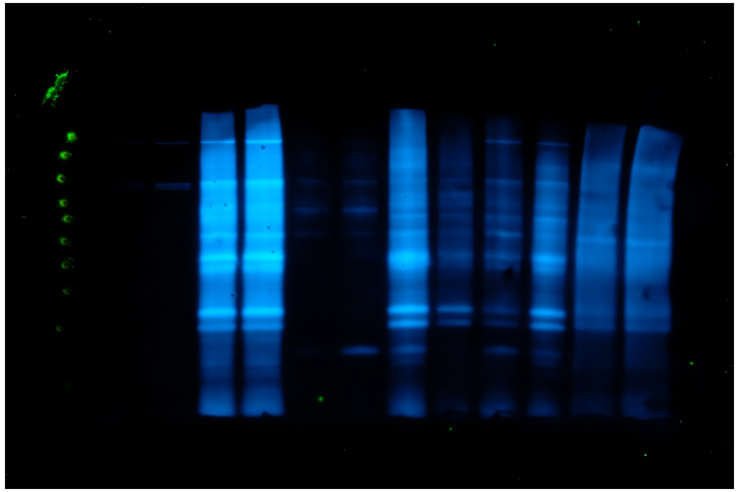

Order (from left to right)

1. TH C99wt before CLICK
2. TH C99mutbefore CLICK
3. Pull-down TH C99wt
4. Pull-down TH C99mut
5. Pull-down ER C99wt
6. Pull-down ER C99mut
7. Pull-down MAM C99wt
8. Pull-down MAM C99mut
9. Pull-down MER c99 wt
10. Pull-down MER C99mut
11. Unbound MAM C99wt
12. Unbound MAM C99mut
